# Exploring the evidence of Middle Amazonian aquifer sedimentary outburst residues in a Martian chaotic terrain

**DOI:** 10.1038/s41598-023-39060-2

**Published:** 2023-10-18

**Authors:** J. Alexis P. Rodriguez, Mary Beth Wilhelm, Bryan Travis, Jeffrey S. Kargel, Mario Zarroca, Daniel C. Berman, Jacob Cohen, Victor Baker, Anthony Lopez, Denise Buckner

**Affiliations:** 1 Planetary Science Institute, 1700 East Fort Lowell Road, Suite 106, Tucson, AZ 85719-2395 USA; 2 External Geodynamics and Hydrogeology Group, Department of Geology, Autonomous University of Barcelona, Bellaterra, 08193 Barcelona, Spain; 3 NASA Ames Research Center, Moffett Field, CA 94035 USA; 4 Department of Hydrology and Atmospheric Sciences, University of Arizona, Tucson, AZ 85721 USA; 5 Blue Marble Space Institute of Science, Seattle, WA 98104 USA; 6 University of Florida, Gainesville, FL 32611 USA

**Keywords:** Planetary science, Hydrology

## Abstract

﻿The quest for past Martian life hinges on locating surface formations linked to ancient habitability. While Mars' surface is considered to have become cryogenic ~3.7 Ga, stable subsurface aquifers persisted long after this transition. Their extensive collapse triggered megafloods ~3.4 Ga, and the resulting outflow channel excavation generated voluminous sediment eroded from the highlands. These materials are considered to have extensively covered the northern lowlands. Here, we show evidence that a lacustrine sedimentary residue within Hydraotes Chaos formed due to regional aquifer upwelling and ponding into an interior basin. Unlike the northern lowland counterparts, its sedimentary makeup likely consists of aquifer-expelled materials, offering a potential window into the nature of Mars' subsurface habitability. Furthermore, the lake’s residue’s estimated age is ~1.1 Ga (~2.3 Ga post-peak aquifer drainage during the Late Hesperian), enhancing the prospects for organic matter preservation. This deposit’s inferred fine-grained composition, coupled with the presence of coexisting mud volcanoes and diapirs, suggest that its source aquifer existed within abundant subsurface mudstones, water ice, and evaporites, forming part of the region’s extremely ancient (~ 4 Ga) highland stratigraphy. Our numerical models suggest that magmatically induced phase segregation within these materials generated enormous water-filled chambers. The meltwater, originating from varying thermally affected mudstone depths, could have potentially harbored diverse biosignatures, which could have become concentrated within the lake’s sedimentary residue. Thus, we propose that Hydraotes Chaos merits priority consideration in future missions aiming to detect Martian biosignatures.

## Introduction

### Background and context

The geologic record of Mars suggests that during the Noachian Period (~ 3.7 Ga to ~ 4.1 Ga^1^), the planet had a surface hydrosphere comparable to Earth's, with extensive fluvial systems, ice sheets, and standing bodies of water^2–6^. However, during the Noachian-Hesperian transition (~ 3.7 Ga), surface hydrologic activity appears to have decreased, and frigid environments became dominant^2^. Observations in Gale crater indicate that this transition may have been gradual^7^.

These extremely cold conditions are believed to have characterized most of Mars' post-Noachian geologic history^2^. However, widespread outflow channels that originate in collapsed highland zones (chaotic terrains) suggest that, while infrequent, catastrophic floods of erupted groundwater led to large-scale landscape modifications^2,8–15^.

The systematic removal of subsurface constituents and the generation of subsurface voids^16^, accompanied by widespread, progressively densifying highland fracturing^17,18^, is likely a fundamental component in chaotic terrain formation. The removal mechanisms implicated in this context are thought to have encompassed an intricate confluence of geological phenomena and processes. These include: (1) hydrological efflux^19^ from water-filled conduits^20^, subsurface lakes^21^, or hydrated evaporites^22^; (2) fluidized, sedimentary outbursts generated by clathrate dissociation^23,24^; and (3) the collapse of volcanic calderas following magmatic drainage^25^. The highland fracturing could have manifested either suddenly and concurrently with the catastrophic flood discharges^8^ or as a component of a protracted terminal subsidence event^16,18^, potentially postdating the abrupt discharge of over-pressurized groundwater^16,18^.

In addition, our knowledge regarding the origins of the outflow channel source aquifers remains incomplete. An early hypothesis, based on observations made by the Viking Orbiter mission, suggested that these aquifers formed part of a global buried hydrosphere that resulted from south polar meltwater infiltration into a lunar-like, impact-generated megaregolith^2,9^. However, this hypothesis lacks explanations for the high-volume, fast discharge rates required for megafloods, as the slow, diffusive flow of groundwater through the megaregolith porous media would have hindered such rapid outbursts.

Furthermore, this scenario proposes that the hydrosphere was confined beneath an ice-rich cryosphere, and that outbursts instigated by regions of gradient-induced overpressures produced the chaotic terrains. However, this theoretical framework fails to satisfactorily rationalize the distinct spatial clustering of outflow channels and chaotic terrains observed in the circum-Chryse region, and their notable absence in other boundary plains regions^14^.

A reconciliatory view is the sourcing of circum-Chryse outflow channels from compartmentalized, elevated aquifers^5,14^. However, the implied magnitude of compartmentalization would have been hard to achieve within a globally extensive megaregolith due to its high permeability^2,9^. Conversely, compartmentalization aligns better with the post-Viking discovery that Martian highlands consist primarily of sedimentary units. For example, higher resolution image datasets from the Mars Global Surveyor (and more recent orbiters) show layering in the highland-forming materials suggestive of a depositional origin^26^. Moreover, the materials forming the highlands include numerous buried impact craters^27^, corroborating their sedimentary origin, which has been hypothesized to have occurred during the pre-Noachian^27^, the Early Noachian^28^ and Middle Noachian^20^.

Analysis of the highland's constituent materials, predominantly characterized by a fine-grained matrix indicative of dust^28^ potentially comprised of clay minerals^29^, suggests a major contribution to the highland stratigraphy from dust fallouts^28^. This extremely ancient dust deposition appears to have stemmed from an atmosphere heavily laden with particulates, a likely byproduct of highly turbulent atmospheric conditions resulting from impact basin formation events during the Early Noachian^28^.

This proposed sedimentary crust, in conjunction with the presence of basaltic layers altered into clays^30^ and evaporite layers such as those in Iani Chaos^31^, could have drastically lowered the subsurface  permeability and acted as hydrological seals or traps.

We propose that the unique physiographic setting of this Martian region, marked by vast chaotic terrains and outflow channels, could be attributed to two factors:

1. The generation of water-filled conduits from converging groundwater zones through the melting of buried icesheets^18,20^ and/or massive evaporite dehydration^16,18,20,22^ confined within these fine-grained, low permeability upper crustal materials.
2. The water-filled conduits' subsequent over-pressurization as their networks intersected an elevated water table that extended from Valles Marineris^20^ to the Tharsis volcanic rise region^19,32,33^. The hypothesized interconnection of these groundwater domains would have facilitated the build-up of the required high hydraulic pressures to generate the massive groundwater outbursts and catastrophic flood discharges.

The enormity of outflow channel sources aligns with the hypothesis that their Late Hesperian floods had sufficient volume to form a northern ocean, and blanket the northern lowlands with thick sedimentary layers^2–5,9,34–36^. Furthermore, while outflow channel formation is thought to have peaked during the Late Hesperian (~ 3.4 Ga)^6,37,^ significantly younger catastrophic floods apparently also occurred during the Early and Middle Amazonian^38,39^. Hence, the total groundwater drained from the circum-Chryse aquifers probably exceeded the volume of the Late Hesperian ocean.

It is important to note that while the discovery of two possible megatsunami deposits associated with possible marine impacts has buttressed the northern ocean case^40,41^, the ocean hypothesis remains disputed by some researchers. For instance, geophysical deformation models are currently incapable of accounting for the extensive elevation variations observed within the proposed shoreline data^42^. Moreover, it has also been suggested that the available Hesperian-era water might be incompatible with the existence of a northern ocean^43^.

### Objectives of the study

The identification of sedimentary units emplaced by fluids released from aquifers is extremely challenging. The outflow channels, being their primary discharge paths, were likely formed through major flood-induced highland erosion. This erosion is thought to have moved immense quantities of material into the northern plains, forming an expansive sedimentary cover^35,37^ over an older lowland Early Noachian basement^27^. Hence, we suggest that mixtures of highland and discharged-groundwater debris likely characterize catastrophic flood deposits. These undifferentiated materials are not suitable geologic targets to seek sedimentary residues of aquifer expulsed fluids.

The northern plains are considered to contain Mars' largest sedimentary unit from outflow channel discharges. The exchange of water between global and regional ice reservoirs may have resulted in the emplacement of younger ice deposits and glacial sediments^44^, further complicating the identification of surface sedimentary units emplaced by catastrophic floods. Additionally, the regional presence of aeolian mantles^10^ adds to this complexity.

**Figure 1:**
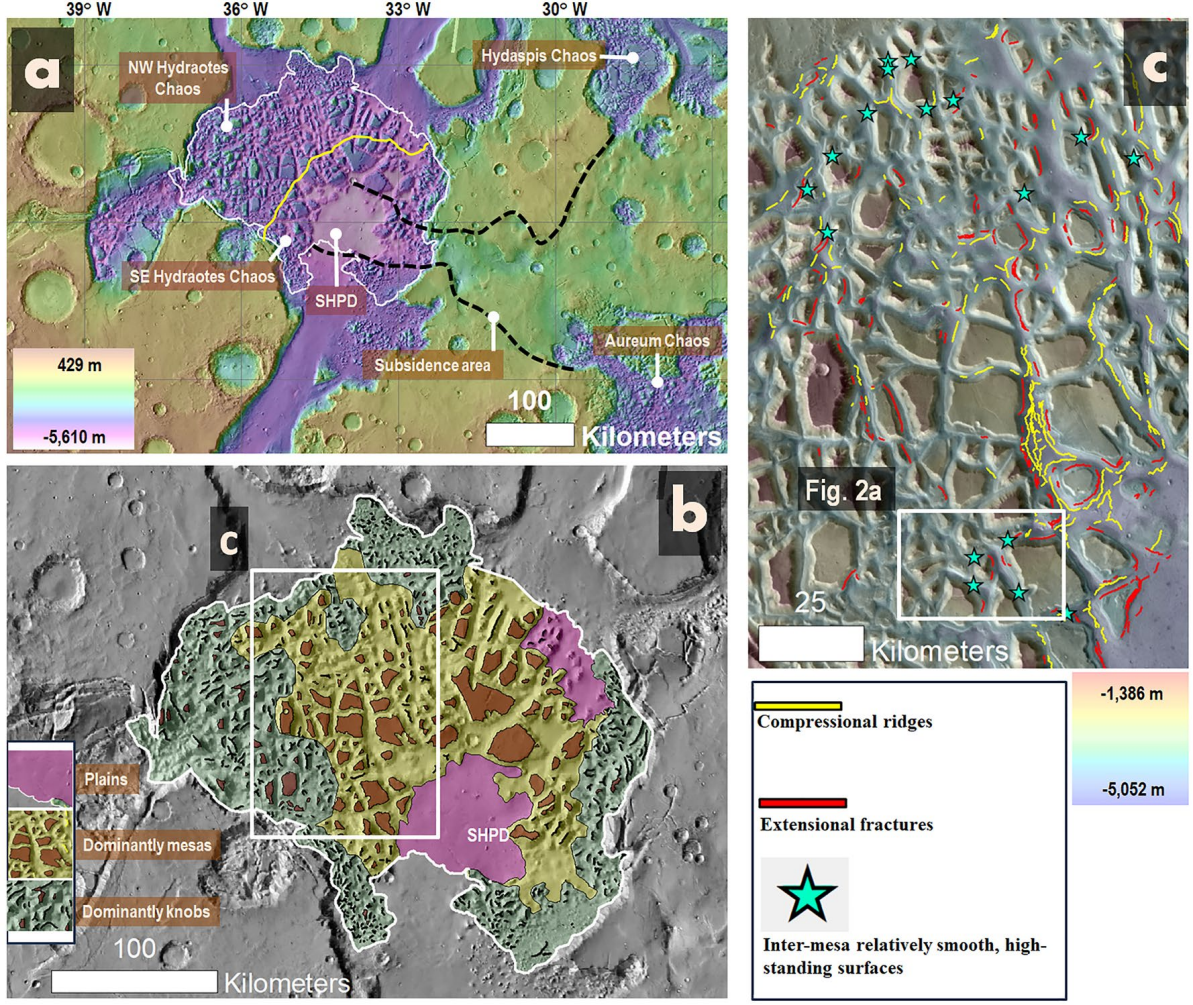
(**a**) A regional view of Hydraotes Chaos (outlined in white) showing the distinct Northwestern (NW) and Southeastern (SE) sections, which include the Southern Hydraotes Plains Deposit (SHPD). The continuity of the SHPD with a subsidence area within the Margaritifer Highlands is also depicted, as evidenced by black lines connecting Hydraotes, Hydapis, and Aureum Chaos. (**b**) A morphological map of Hydraotes Chaos showing the predominant plains, mesas, and knobby regions. (**c**) A more detailed morphological map of a section of Hydraotes Chaos, highlighting potential extensional fractures, contractional ridges, and smooth surfaces that bound topographically depressed inter-mesa floors. (**a**, **b**) The map base blended HRSC-MOLA DEM overlying a shaded relief (200 m/pixel, credit: MOLA data—NASA, HRSC data—ESA/DLR/FU Berlin). (**c**) HRSC-MOLA DEM (200 m/pixel, credit: MOLA data—NASA, HRSC data—ESA/DLR/FU Berlin) over a THEMIS daytime IR global layer (http://www.mars.asu.edu/data/, 100 m/pixel, credit: Christensen, et al.^147^). We produced this figure using Esri's ArcGIS 10.3 (http://www.esri.com/software/arcgis).

Our objective is to identify sedimentary deposits that were expelled from aquifers and deposited close to the discharge outlets. We focus on Hydraotes Chaos (Fig. 1a,b), a chaotic terrain in southern circum-Chryse with widespread terraced polygonal mesas. Hydraotes Chaos has been interpreted to have been the deepest part of a catastrophic-flood generated, Late Hesperian (~ 3.4 Ga) inland sea contained within a vast basin in southern circum-Chryse^45^, which subsequently regressed to form a paleolake within the chaotic terrain^46^. Our results indicate that, ~ 2.3 Ga later, a regional aquifer regionally discharged forming a mud lake, which ponded within the chaotic terrain’s deepest enclosure. We consider the mud to be dominantly ‘clay’ grain size, probably with a phyllosilicate-rich composition^29,30^.

## Geological analysis methods

**Figure 2:**
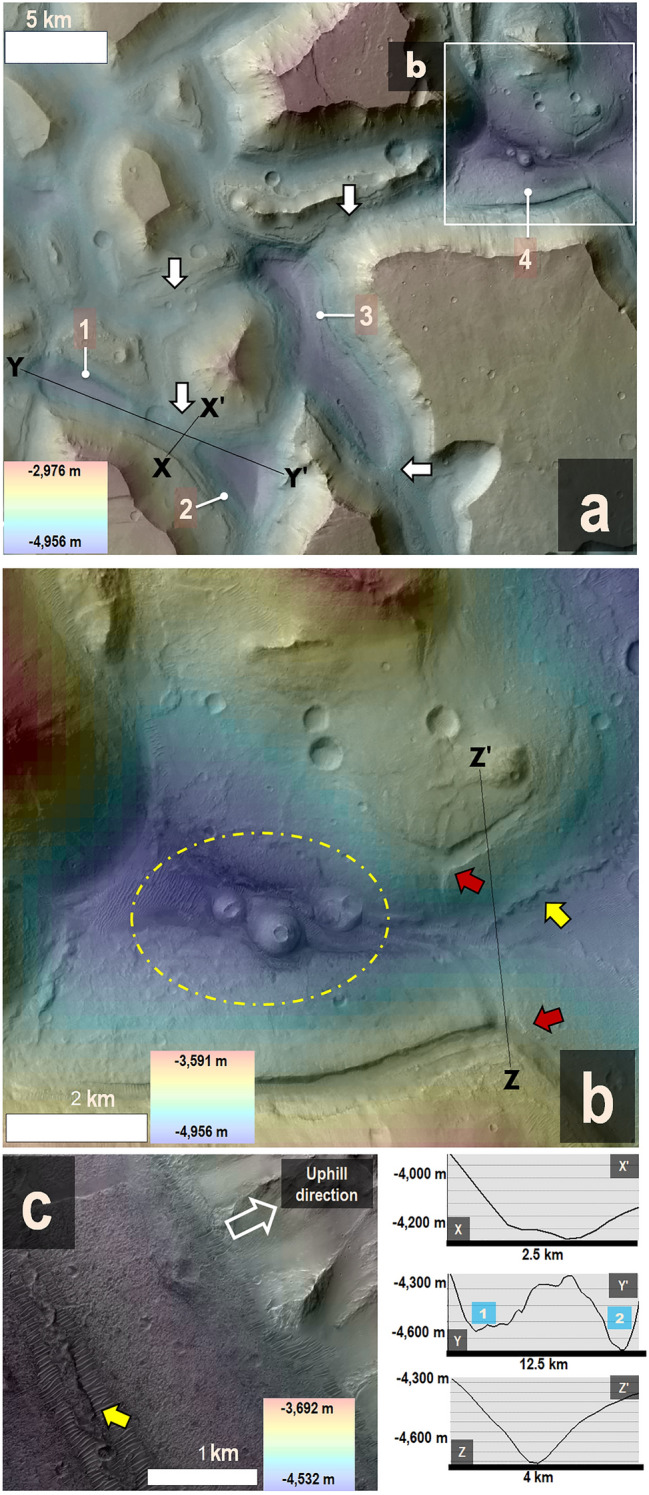
(**a**) Close-up view of Fig. 1c, where the white arrows point to the regional smooth floor sections with a base elevation close to approximately − 4200 m (for example, elevation profile X to X′). The numbers 1–4 mark the locations of neighboring enclosed depressions, which have bases at approximately − 4700 m (as shown in elevation profiles Y to Y′, Z to Z′). (**b**) A closer look at a portion of panel (**a**), specifically one of the enclosed depressions. Red arrows highlight Y-shaped fracture patterns, suggesting extension and subsidence related to the origin of the depressions. The yellow arrow points to a sinuous, uphill-facing scarp, which is consistent with compression along the convergence zones of opposing subsided flanks. A dashed, yellow ellipse encloses pitted cones within the subsided depression. (**c**) An even closer view of a compressional ridge, showing that it has an uphill-facing scarp (yellow arrow). (**a**, **b**) HRSC and MOLA DEM (200 m/pixel; MOLA data credit: NASA; HRSC data credit: ESA/DLR/FU Berlin) superimposed on a portion of a CTX mosaic (6 m/pixel; credit: NASA/JPL/Malin Space Science Systems^48^) (*Source*: https://www.msss.com/mro/marci/images/tips/mediatips.html). (**c**) HiRISE image ESP_071490_1805, with ~ 50 pixels per degree, centered at 1° 0′ 10.74″ N; 34° 33′ 36.05″ W (credit: NASA/JPL/University of Arizona) (*Source*: https://www.uahirise.org/media/usage.php).

**Figure 3:**
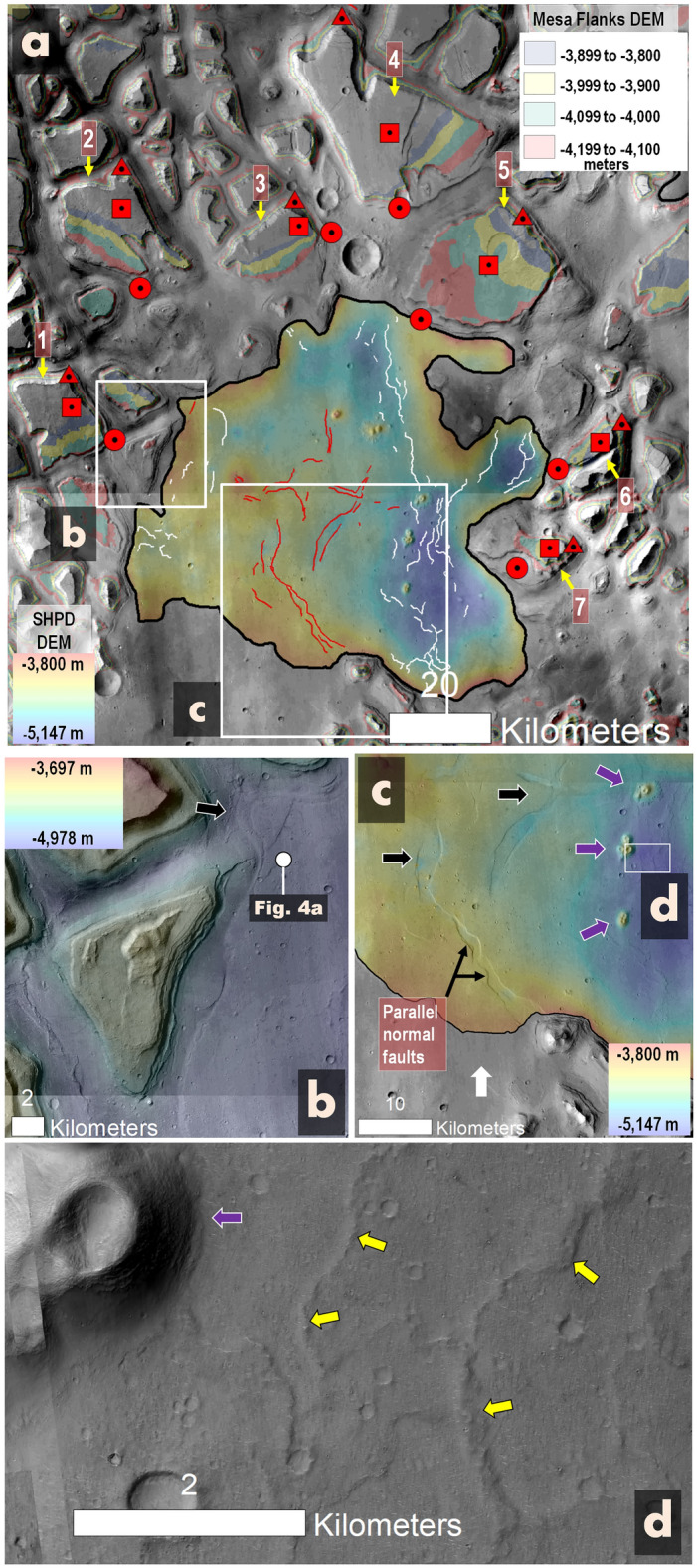
(**a**) This view of southern Hydraotes Chaos highlights key features of the SHPD, including its uniform elevation margin (black outline), internal grabens (red lines), wrinkle ridges (white lines), and seven distinct, rotated mesas (red squares, labeled 1–7). Margins of these mesas tilting away from the SHPD (red triangles) exhibit terraced features within the − 3800 m to − 4100 m elevation range, as detailed in Fig. S1. Margins tilted towards the deposit (red circles) are lower elevations, as detailed in Fig. S2. (**b**) Close-up of the SHPD's western part shows a lobate margin (black arrow) over a subsidence-reshaped floor. The location of Fig. 4a is indicated. (**c**) Detailed view from panel (**a**) presents grabens (black arrows) and potential mud volcanoes (purple arrows). (**d**) Zoomed-in view from panel (**c**) highlights potential mud volcanoes lacking adjoining mud breccias (purple arrow), and potential wrinkle ridges (yellow arrows). (**a**–**c**) HRSC and MOLA DEM (200 m/pixel; credit: NASA for MOLA data, ESA/DLR/FU Berlin for HRSC data) overlaid on sections of a CTX mosaic (6 m/pixel; credit: NASA/JPL/Malin Space Science Systems; reference^48^) (source: https://www.msss.com/mro/marci/images/tips/mediatips.html). (**d**) A portion of a CTX mosaic (6 m/pixel; credit: NASA/JPL/Malin Space Science Systems; reference^48^) (source: https://www.msss.com/mro/marci/images/tips/mediatips.html).

**Figure 4:**
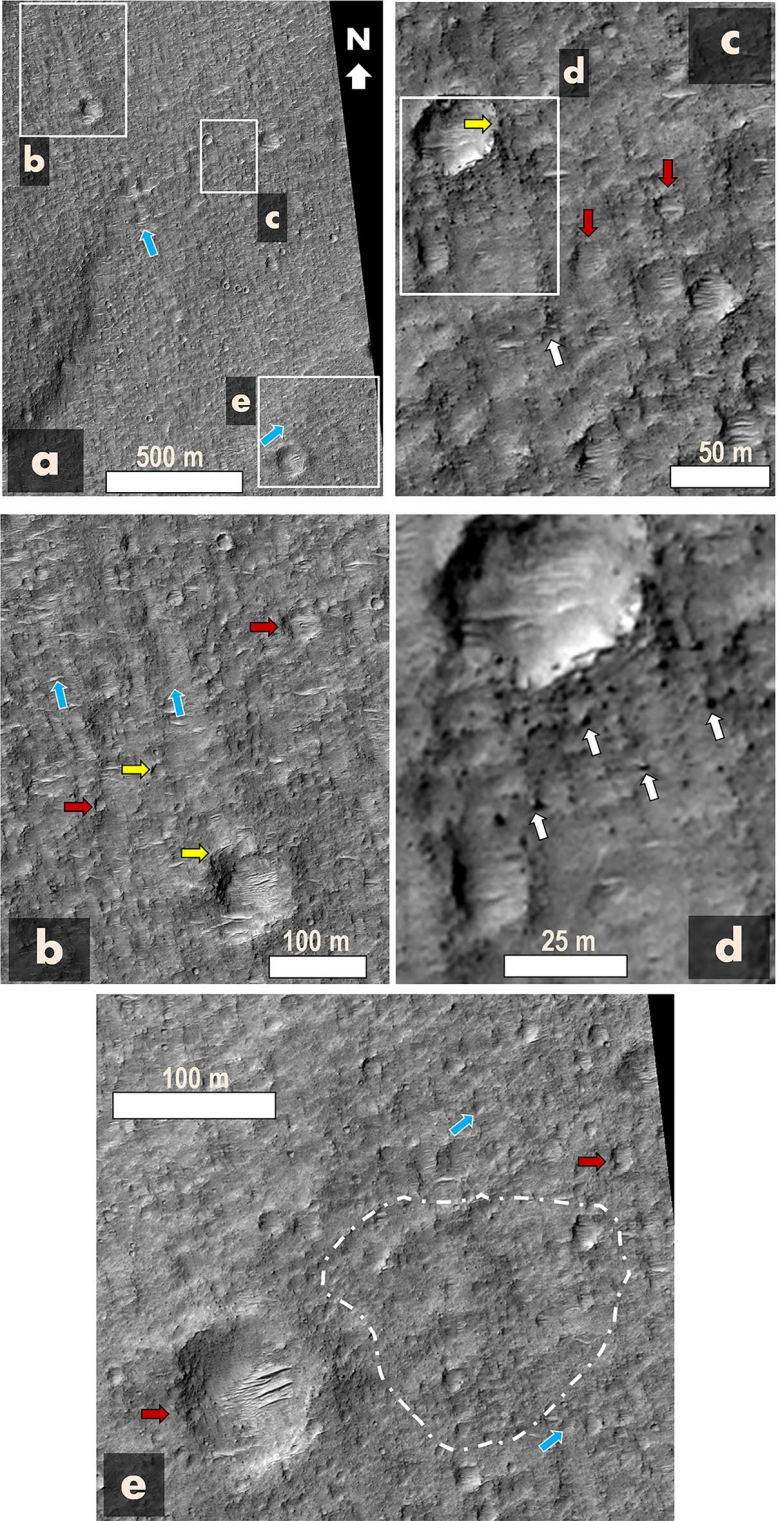
Detailed analysis of the SHPD's lobate margin pinpointed in Fig. 3b, as captured in HiRISE image ESP_071490_1805 with approximately 50 pixels/degree. Key geological features are highlighted: blue arrows direct attention to scour marks trending northwest and northeast, depicted by closely spaced grooves and ridges (panels **b**–**d**). The grooves and ridges likely indicate the erosional effects from crosswinds. Within some of these ridges are boulder piles, suggestive of localized aeolian deflation, as highlighted by white arrows in panel (**d**) and (**e**). The yellow arrows in panels (**b**) and (**c**) emphasize the truncation of various craters by the grooves. Red arrows in panels (**b**–**e**) denote the widespread presence of degraded craters with diameters ≤  ~ 100 m, with some further eroded into somewhat irregular and apparently flat-rimmed or rimless depressions at certain locations, as demonstrated within the zone bordered by the dashed white line in panel (**e**). Note that although the arrowheads in panel (**e**) end at pits, their primary role is to emphasize the lineated spatial trends they indicate. Image credit: NASA/JPL/University of Arizona Usage policy.

**Figure 5:**
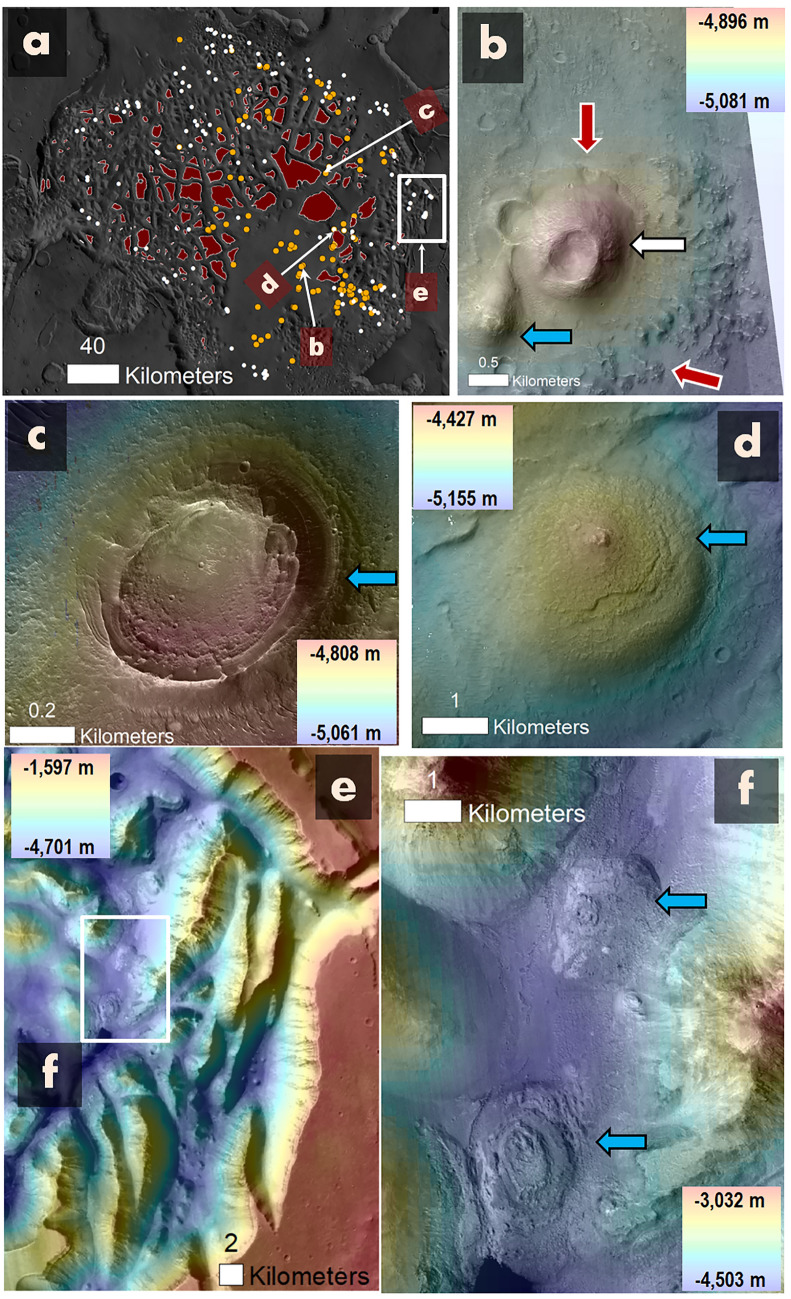
A series of observations showing the potential existence of mud volcanoes and diapirs. (**a**) Morphological map highlighting possible mud volcanoes (orange dots) and diapirs (white circles), with their distribution limited to the plains and inter-mesa surfaces and absent on the mesas (red-shaded areas). (**b**) Detailed view of a pitted cone (potential mud volcano, white arrow) adjacent to a probable eruptive deposit with retreat margins extending to the cone's edges (red arrows). An adjacent dome-like structure, possibly a diapir, is denoted by the blue arrow. (**c**) A proposed eroded diapir potentially exposing finely layered strata that have been tilted upwards during buoyant ascension (blue arrow). (**d**) Another possible diapir forming a dome of uplifted floor materials (blue arrow). (**e**) View of eastern Hydraotes Chaos, displaying breakaway ridges and troughs, potentially exposing deep highland stratigraphy. (**f**) Detailed image from panel (**e**), pinpointing potential diapirs (blue arrows). (**a**) The map's base is an overlaid HRSC-MOLA DEM on a MOLA shaded relief (200 m/pixel; credit: MOLA data—NASA, HRSC data—ESA/DLR/FU Berlin). (**b**) An HRSC-MOLA DEM (200 m/pixel; credit: MOLA data—NASA, HRSC data—ESA/DLR/FU Berlin) overlaid on a section of a CTX mosaic (6 m/pixel; credit: NASA/JPL/Malin Space Science Systems; reference^48^) (source: https://www.msss.com/mro/marci/images/tips/mediatips.html). (**c**) A CTX stereopair-derived DEM overlaid on a section of a CTX mosaic (6 m/pixel; credit: NASA/JPL/Malin Space Science Systems; reference^48^) (source: https://www.msss.com/mro/marci/images/tips/mediatips.html). (**d**–**f**) An HRSC-MOLA DEM (200 m/pixel; credit: MOLA data—NASA, HRSC data—ESA/DLR/FU Berlin) overlaid on a section of a CTX mosaic (6 m/pixel; credit: NASA/JPL/Malin Space Science Systems; reference^48^) (source: https://www.msss.com/mro/marci/images/tips/mediatips.html).

**Figure 6:**
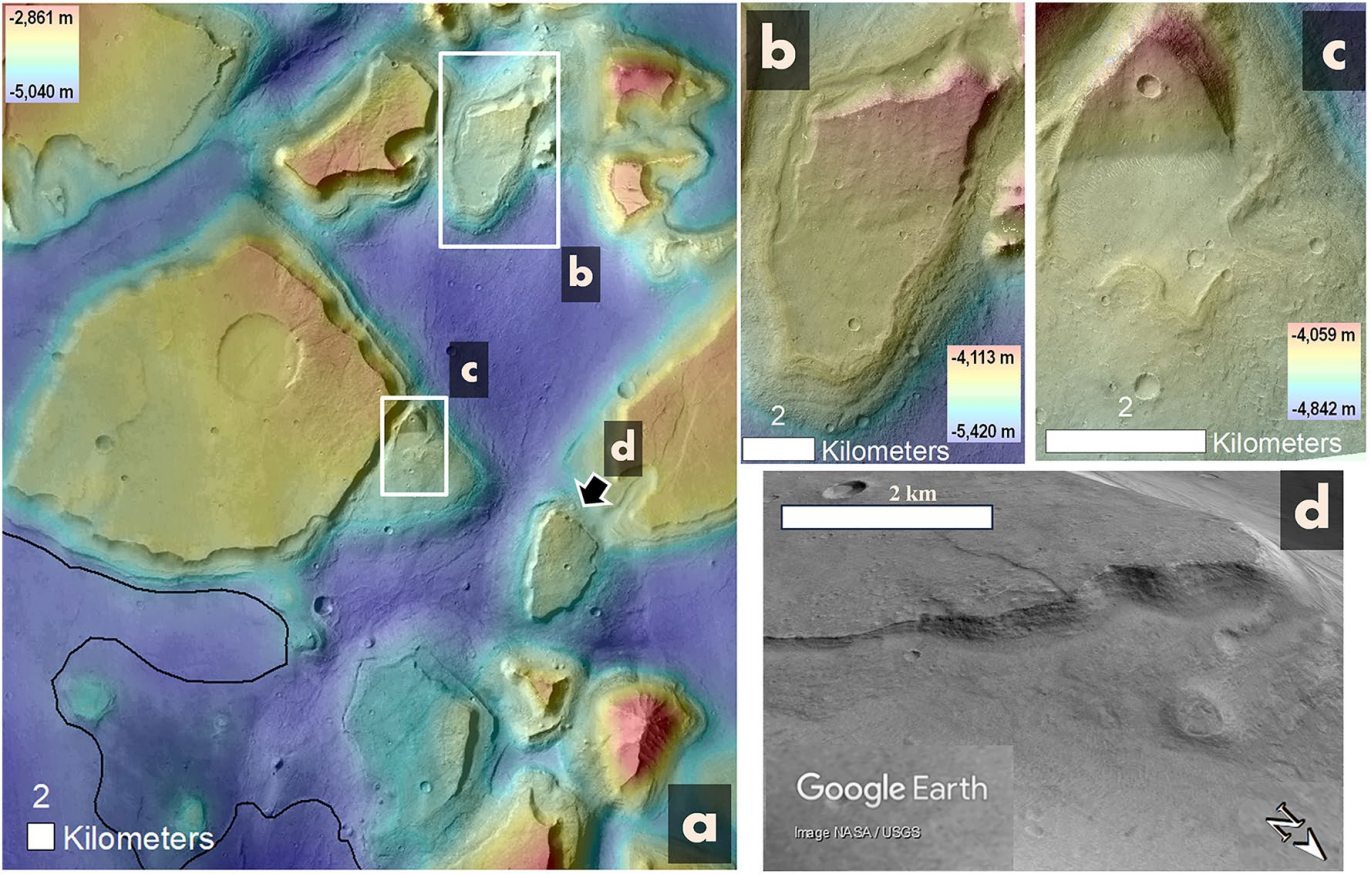
(**a**) A view of a section of southern Hydraotes Chaos. The black line delineates the regional margin of the hypothesized mud lake residue. Panels (**b**), (**c**), and (**d**) highlight examples of mesas with inclined surfaces that seem to be buried under floor-forming materials exhibiting local lobate patterns (white arrows). Specifically, panel (**d**) provides a perspective view, allowing observation of a mesa flank gradually becoming buried by the adjacent floor materials (Credit: Google Earth). Panels (**a**) to (**c**) utilize an overlaid HRSC-MOLA DEM on a portion of a CTX mosaic (200 m/pixel and 6 m/pixel respectively; credits: MOLA data—NASA, HRSC data—ESA/DLR/FU Berlin, CTX mosaic—NASA/JPL/Malin Space Science Systems; reference^48^) (source: https://www.msss.com/mro/marci/images/tips/mediatips.html).

**Figure 7:**
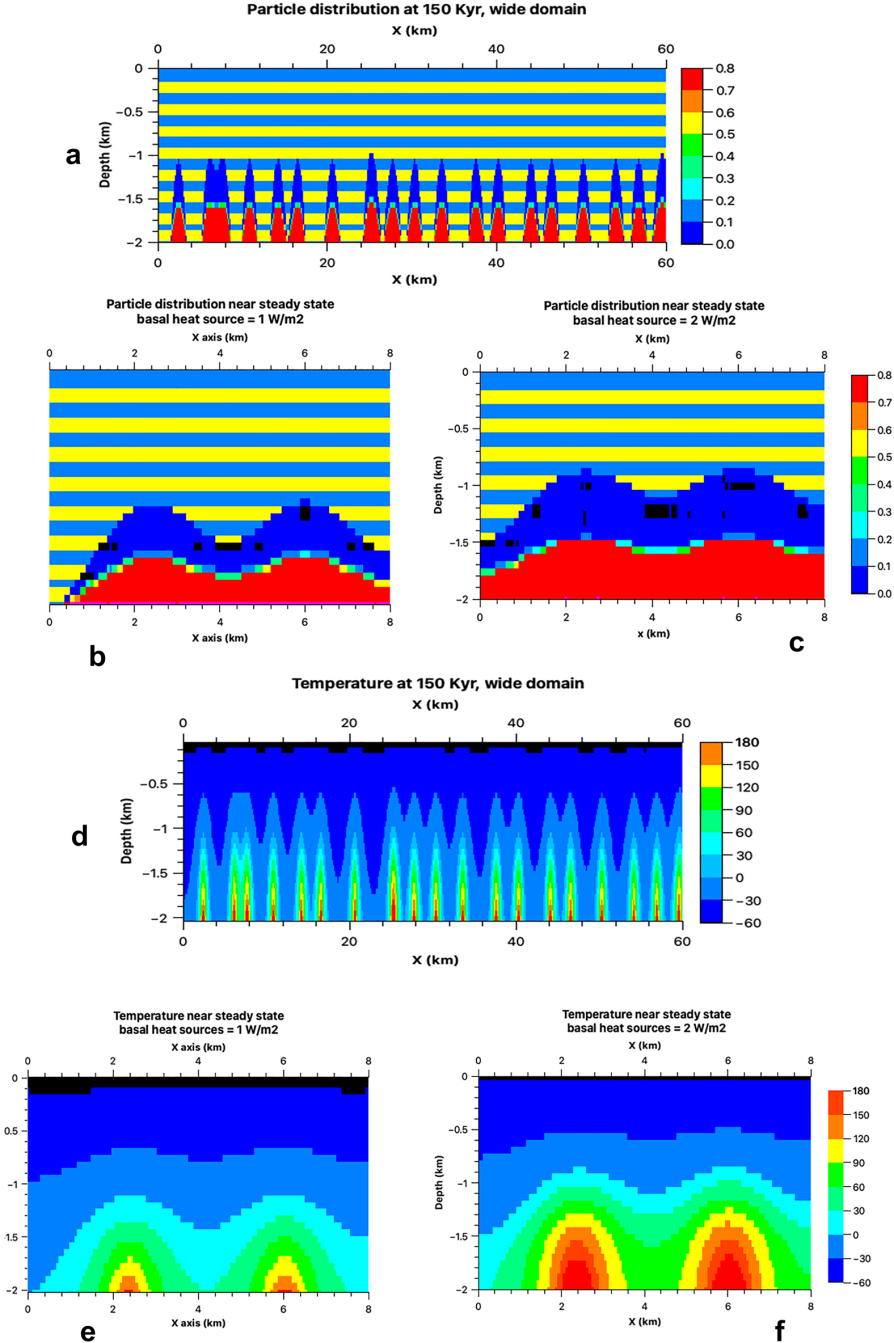
(**a**) Broad view of the distribution of the total particulate load at 150 Ka. The igneous intrusions are randomly distributed with an average spacing of ~ 3.5 km. (**b**–**c**) Close-up views at 300 Ka, with panel (**c**) showing a higher heat input scenario. An approximate steady state is reached before 300 Ka. Blue and yellow stripes represent frozen mud layers with high and low particulate content, respectively. In the melted region, particles have settled to form a porous bed (depicted in red) with cumulative thicknesses typically ranging between ~ 300 and 500 m. Above this bed are the water chambers, with thicknesses varying from ~ 400 to 650 m for the two heating rates presented. (**d**, **e**) Depictions of temperature distribution in corresponding model domains at a heating rate of 1 W/m^2^. (**f**) Temperature distribution at a heating rate of 2 W/m^2^. Most of the vertical structure develops by 150–200 Ka; after this period, growth is primarily lateral, resulting in the connection of individual water chambers.

We produced a detailed geologic investigation of Hydraotes Chaos based on image and topographic data analysis (Figs. 1, 2, 3, 4, 5, 6) as well as sophisticated numerical simulations (Fig. 7).

### Tools and datasets

We conducted our morphologic mapping using Esri's ArcGIS Desktop 10.3 (http://www.esri.com/software/arcgis). We utilized the publicly available global seamless Mars Reconnaissance Orbiter (MRO) Context Camera (5.6 m per pixel, CTX)^47^ visible light mosaic from the Murray Lab^48^ (http://murray-lab.caltech.edu/CTX/), Mars Odyssey Thermal Emission Imaging System (100 m per pixel, THEMIS)^49^ nighttime and daytime infrared image mosaics^50^, in combination with Mars Global Surveyor (MGS) Mars Orbiter Laser Altimeter (MOLA)—Mars Express (MEX) High-Resolution Stereo Camera (HRSC) blended Digital Elevation Model (200-m-per-pixel DEM)^51^. We also produced some meter-scale to decameter-scale characterizations using images from the Mars Reconnaissance Orbiter's High-Resolution Imaging Science Experiment (0.25 to 1.3 m per pixel, HiRISE)^52^.

### Geomorphological mapping approach

Hydraotes Chaos includes a vast number of mesas and knobs. Mesas are generally polygonal and flat topped (sometimes inclined), whereas knobs are generally smaller than mesas, mainly have rounded bases seen in plan view and have conical or rounded mound-shaped profiles. Some knobs may also have summit craters.

The geomorphologic map displayed in Fig. 1a was produced using a CTX base layered beneath a semi-transparent MOLA-HRSC DEM. The mapping process was performed manually at a scale of 1:100,000 to ensure accuracy and detail. The surface edges of most mesas were traced as polygons, facilitating a detailed representation of their structure. Similarly, we captured a large subset of ridge-forming knobs, focusing particularly on those with clear crests, by representing them as polylines. In addition, the margins of two extensive plains units were traced as polygons, offering a thorough illustration of their extents. Lastly, to prevent cluttering and enhance practicality, we constructed two base map polygons, each enclosing areas dominated either by mesas or knobs. This approach allowed for an efficient and clear representation of these dense features on the geomorphologic map.

The morphologic maps in Figs. 2c, 3a, 5a were generated at a mapping scale of 1:30,000 using a CTX base under a semi-transparent MOLA-HRSC DEM. The mapping process was conducted manually, utilizing polylines to denote fractures (extensional faults) and compressional ridges. Points were employed to mark potential diapirs and mud volcanoes. The mesa mapping shown in Fig. 5a was adapted from Fig. 1a. Points were also used to highlight the locations of terraced mesas tilted towards the Southern Hydraotes Plains Deposit (SHPD), as well as the positions of both lowered and non-lowered margins.

### Numerical simulation setup

We conducted our simulation using MAGHNUM_PRTCLS, an open-source toolset that has been previously used for similar purposes^53–56^.

#### Numerical simulation geological foundations

The following constraints are based on our results (presented later in this article) and previous, referenced research. They include the following two assumptions:

*Role of intrusive magmatism in the middle Amazonian resurfacing phase of Hydraotes Chaos.* The southern circum-Chryse outflow channel record includes evidence of a possible warm paleoclimatic spike during the Middle Amazonian^38,39^. The presence of Amazonian-aged fluvial features^57–59^, alluvial fans^60^, deltas^61,62^, and glacial landforms^63^ also supports the development of transient warmer and wetter Amazonian conditions. Hence, it is possible that this paleoclimatic anomaly produced top-down propagating thermal waves within the inter-mesa regions, which upon reaching the icy mudstone, facilitated its subsequent melting, solid–liquid phase differentiation, and aquiferous chamber formation.

However, the inferred sequence of events involving subsidence and aquifer releases is inconsistent with recurrence (detailed in the results section). In contrast, if driven by paleoclimatic cyclicity, which could have been part of the previously hypothesized anomaly, these processes could have produced early subsidence phases and material extrusion that would be disturbed by subsequent cycles. Additionally, if a paleoclimatic cause were responsible, we would expect comparable top-down thermal waves across similar latitude and elevation ranges, leading to significant landscape changes over these broader regions. However, the geomorphology that we have documented is exclusively confined to the chaotic terrain floor, having played a crucial role in creating its intricate current topography.

Hence, we infer that factors beyond paleoclimatic influence were at play. We propose a magmatic thermal trigger as the underlying mechanism for the complex topography observed in the chaotic terrain, as presented in our study.

*Utilizing mudstones as the base stratigraphy in the simulation.* In our simulation, we used mudstones as the foundational stratigraphy, a decision backed by evidence that the possible mud lake residue within the chaotic terrain is probably a fine-grained sedimentary unit. Numerous studies also suggest that fine-grained deposits constitute significant portions of the highland-forming materials^28,30^. Furthermore, we incorporated high ice content layers into the mudstone stratigraphy. These ice layers, similar in composition to mid-latitude ice sheets as documented by Piqueux et al.^64^, could have been episodically emplaced throughout a gradual highland construction history^18,20^.

#### Boundary conditions

We implemented the following boundary conditions:

1. We utilized a 2-km thick domain for our simulation, structured with square computational cells. Vertically, there were 32 cells, together accounting for a 2-km depth. For the narrow domain, we used 128 cells horizontally, giving it a total horizontal length of 8 km. Hence, each computational cell was 62.5 m × 62.5 m. For the wide domain simulation, we specified a grid that was 960 cells wide, covering a horizontal span of 60 km.
2. Our numerical simulation incorporated a stratigraphic model of the subsurface, featuring periodic sequences of water ice-enriched mudstone and unlithified sedimentary deposits. The strata were designed to be 100–120 m in thickness, adhering to the approximate stratigraphic continuity and scale observed in Fig. 6d. The mud layers were characterized by high porosity (35%) and were saturated with H_2_O ice. In contrast, the unlithified sedimentary layers exhibited lower water ice content and moderate porosity (25%) but were also H_2_O ice saturated. We assumed spatial homogeneity across these layers, acknowledging that potential discontinuities could give rise to areas undermined by meltwater adjacent to meltwater-free zones. The permeability of these layers was not considered a significant factor, given that layers fully saturated with H_2_O ice verge toward zero permeability. Upon the onset of melting, the flow was assumed to transition into a continuum-like state due to the disintegration of the layered materials when they become wet and unstressed. This leads to the gradual growth of water-filled chambers and the formation of a porous bed at the bottom as particles descend. For situations where permeability is of concern, the mudstone porosity–permeability relationship documented by Yang and Aplin^65^ offers a valuable reference.
3. The layers in our simulation contained lithic particles with sizes ranging from 5 cm to 1 micron, adhering to a fractional distribution typical of mudstone. The physical properties assigned to these particles included a grain density of 2700 kg/m^3^, thermal conductivity roughly equivalent to 1.6 W/m/K^66^, and a specific heat capacity of 840 J/kg/K.
4. Properties of H_2_O, liquid or frozen, (density, specific heat, enthalpy, latent heat of melting, viscosity) as a function of temperature and pressure are from SUPCRT92^67^.
5. The geothermal heat flux through the bottom boundary in our simulation was set at 40 mW/m^2^. It is important to note that heat flux varies over the surface of Mars, and historical estimates show a range from about 100 mW/m^2^ during the Noachian era to approximately 10–30 mW/m^2^ in the present day^68^. Our chosen value of 40 mW/m^2^ represents a reasonable approximation for Mars around 2–3 Ga.
6. For our simulation, we used the gravitational acceleration of Mars, which is 3.7 m/s^2^.
7. The initial conditions were set with a surface temperature of − 63 °C, while the base temperature was established at − 13 °C, 2 km below the surface.
8. To represent magmatic heating, we imposed a high heat flux of 1 W/m^2^ along the bottom boundary at two discrete segments. These segments were each approximately 300 m long, centered at 2.1 km and 6 km (3.9 km spacing), respectively, along the base of the 8 km-wide computational domain. In a broader domain simulation, as illustrated in Fig. 7a, d, we extended the domain width to 60 km. Here, intrusive heating at the base was randomly spaced with an average spacing of 3 km. This configuration suggests that dike intrusions could have served as potential heat sources. The computed spacing of 3 to 3.9 km is broadly analogous to the topographic segmentation, which we interpret as subsidence in this article. This is exemplified by the ~ 5 km spacing observed in the elevation profile from Y to Y′ as seen in Fig. 2.
  The size of subsurface aquiferous chambers hinges on factors like geothermal heat flux, surface temperature, and host material properties. These are represented by the thermal diffusivity parameter, which, through its square root, governs the temperature distribution within the subsurface. However, among these variables, the heat from magmatic intrusions emerges as the most critical factor. It is this heat injection that primarily dictates the size of the chambers, effectively superseding the influence of other variables such as the geothermal gradient.
9. During the melting phase, our simulation monitors the volume-based size distribution of lithic particles, initially embedded in the layers. Particles smaller than 1 μm remain indefinitely suspended in the liquid water, leading to a turbid or 'muddy' mixture. The flow of these particles during this phase is governed by Stokes' law. Although size distributions for mudstones can vary, they typically contain approximately 30% clay (less than 0.005 mm), 50% silt (less than 0.125 mm), and 20% sand (less than 2 mm)^69,70^.

## Geomorphological analysis of Hydraotes Chaos

We identified three broad geomorphological units within Hydraotes Chaos, consisting of polygonal mesas, knobby fields, and extensive plains (Fig. 1b). The plains include the Southern Hydraotes Plains Deposit (SHPD) (Fig. 1b), which aligns with a broad curvilinear depression within the adjacent highlands to the east (Fig. 1a).

There are widespread inter-mesa floor sections (e.g., star symbols in Fig. 1c, white arrows in Fig. 2a), which consistently exhibit elevations around − 4200 m (e.g., elevation profile X to X′ in Fig. 2). These inter-mesa floors abruptly transition into enclosed depressions, contributing to a highly segmented topography (numbers 1–4 in Fig. 2b, elevation profiles Y to Y′ and Z to Z′ in Fig. 2). The depressions exhibit fractured margins, including Y-shaped fracture patterns, or triple junctions (red arrows in Fig. 2b), as well as sinuous ridges on their floors (yellow arrows in Fig. 2b,c).

Numerous terraces flank the mesas at consistent elevations ranging between roughly − 3,800 m and − 4200 m (Fig. S1). Interestingly, terrace sections oriented away from the SHPD maintain this elevation range across the southeastern part of the chaotic terrain (Numbers 1–7, red triangles in Fig. 3a). In contrast, those facing the SHPD are situated at lower elevations (Numbers 1–7, red circles in Fig. 3a). These mesas also exhibit consistent surface tilt components leaning towards the SHPD (Fig. S2).

The SHPD is demarcated by a margin with an approximately constant elevation of ~ − 4800 m (Fig. 3a). The marginal materials locally overlap mesa-flanking floor depressions (Fig. 3b). The deepest areas of the SHPD are found on its western extents, reaching ~ 350 m below the margin (Fig. 3a). These deep areas are bounded by broad grabens (Fig. 3c) and include low-lying sinuous ridges (Fig. 3a,d).

The close examination of the lobate margin of the SHPD identified in Fig. 3b reveals widespread scour marks displaying patterns trending towards the northwest and northeast (blue arrows in Fig. 4). These patterns are characterized by closely spaced grooves and ridges (Fig. 4b–d), some apparently composed of boulder piles (white arrows in Fig. 4c,d). In addition, many of the grooves truncate numerous craters (e.g., yellow arrows in Fig. 4b,c). The examined lobe’s area includes widespread degraded craters at diameters ≤  ~ 100 m (e.g., red arrows in Fig. 4), including some modified into irregular flat-rimmed (or no-rim) depressions (e.g., zone outlined by dashed white line in Fig. 4e).

Pervasive pitted cones occur throughout the trough and SHPD floors (Figs. 2b, 5a, white arrow in Fig. 5b). In addition, the trough floors also exhibit abundant dome-like structures (Fig. 5a, blue arrows in b–d). However, the chaotic terrain’s mesa surfaces are void of these features (Fig. 5a). Some of the pitted cones retain encircling deposits flanked by irregular scarps, which extend to the cones' margins (red arrows in Fig. 5b). Some of the domical structures consist of fractured elevated areas (blue arrow in Fig. 5c), while others are surrounded by inward-dipping unconformities around concentric layers (blue arrow in Fig. 5d). Furthermore, some domes are observed to host mesas atop them, with these mesas exhibiting inclined surfaces that extend towards adjacent plains areas encircled by lobate margins (Fig. 6).

## Integrated interpretation of findings

### Evidence of middle Amazonian evacuation of fluidized mud

We posit a post-Hesperian resurgence of subsidence, instigating a stark morphologic dichotomy (yellow line in Fig. 1a); this process affected the northwest inter-trough floors and most of the chaotic terrain's southeast region.

The northwestern region subsidence modifications are substantiated by depressions with extensionally fractured margins (e.g., Figs. 1c, 2b), partitioning the topography of the trough floors between mesas (Fig. 2). In particular, Y-shaped fracture patterns, or triple junctions, commonly denote localized subsidence in the surrounding areas, giving the intersection point an apparent relative elevation (red arrows in Fig. 2b). The radiating arms of this Y-structure are a consequence of tensional stresses causing the ground to fracture into extensional faults. Furthermore, the presence of sinuous uphill-facing scarps along the lower floor areas (e.g., yellow arrows in Fig. 2b,c) is consistent with surface creep and thrusting associated with gravitational spreading processes during subsidence^71^.

In the southeastern region, there are several terraced mesas leaning towards their neighboring SHPD flanks (Fig. 3a), indicating that subsidence extended beyond the troughs and was centered within the SHPD. The SHPD's near-uniform elevation margin (Fig. 3a) alludes to a ponding origin. Its overlap with adjacent inter-mesa, subsided floors (Fig. 3b) implies that ponding happened post-subsidence, yet likely related to it.

We propose that the observed subsidence patterns suggest extensive subsurface material depletion, largely offset by substantial outflows forming a paleolake in the chaotic terrain's southeast. Consequently, we propose that SHPD is this paleolake’s residue. We conducted a count of impact craters on its surface. This analysis allowed us to estimate that this deposit formed during the Middle Amazonian, ~ 1.1 Ga (Fig. S3), implying subsurface releases ~ 2.3 Ga after the regional Late Hesperian catastrophic floods had ceased^6^.

High-resolution SHPD close-up views reveal extensive NW and NE lineated patterns, including grooves, which locally truncate crater margins (e.g., yellow arrows in Fig. 4b,c). Graben-horst systems, resulting from tectono-volcanic activity, can give rise to similarly structured topography.

However, the regional presence of widespread, highly eroded craters with diameters smaller than 100 m (generally 30 m deep or less) cannot be attributed to ancient tectonic forces, suggesting that wind erosion was determinant in shaping the lineated patterns (Fig. 4). Additionally, the presence of bouldery ridges amidst scoured terrains attests to wind-induced deflationary processes, characterized by selective removal of fine sediments and consequent emergence of residual boulders (white arrows in Fig. 4c,d).

We observe no typical pahoehoe or ‘a‘ā textures, suggesting that lava flows probably did not significantly contribute to the SHPD’s emplacement. Hence, we suggest that this terrain consists of predominantly fine-grained material, suggesting that the paleolake likely ponded a muddy fluid.

We propose that the SHPD was expulsed from a buried layer through powerful sedimentary outflows—while generating pervasive subsidence accompanying tilting of mesas and other shallow rooted tectonics—erupting as a sediment-rich fluid to form a mud lake. In a similar manner, but inferred from a rheological model, Wilson and Mouginis-Mark^72^ interpreted that a distinctive flow deposit southwest of Cerberus Fossae was a subsurface released mud flow.

The deepest SHPD areas, reaching ~ 350 m below its margin (Fig. 3a), are flanked by broad grabens (Fig. 3c), suggesting that after its initial ponding, its surface solidified and experienced extensional stresses due to subsidence as its subsurface lost voluminous volatile content. Low-lying wrinkle ridges on these deep SHPD areas (Fig. 3a,d) are consistent with compressional stresses concentrated close to the convergence areas, compensating for the lateral extension during the subsidence. The proposed large scale of subsidence affecting the solidified mud lake’s surface suggests the retention of large water volumes within the sedimentary outflows that generated the mud lake.

Surface fractures reaching the water-rich mud lake residue could have readily driven its volume loss within the deposit through sublimation, evaporation, or fluidized emanations. An analog consideration is the drainage of a sub-ice lake on the Amery Ice Shelf in East Antarctica in mid-winter 2019, which resulted in the ice doline (a significant depression in the surface of the ice shelf^73^). This event, caused by the loss of approximately 600–750 million cubic meters of sub-ice water, resulted in a lowering of the local surface elevation by an average of 24 m, with the lowest point reaching 80 m^73^.

### Geothermal processes generating aquiferous chambers within ice–rich mudstone stratigraphy

We propose that the segmented subsidence within the NW Hydraotes Chaos inter-mesa floors reflects the destabilization of interconnected, closely spaced aquiferous chambers that developed within an ice-rich stratigraphy.

The overarching concept of phase segregation, posited as the cause for Martian interconnected aquiferous chambers, has previously been suggested as an explanation for the notably extensive subsidence patterns observed in the curvilinear formations within the highland regions located between Aromatum and Hydaspis Chaos (Fig. 1a). This theoretical framework was initially presented by Rodriguez and colleagues in their 2005 and 2015 publications^16,18,20^.

To explore the plausibility of melt segregation hydrodynamics giving rise to chains of aquiferous chambers, we executed advanced numerical simulations (Fig. 7). The results indicate that when magmatic heat is applied to the ice-rich mudstone stratigraphy, the ice melts, releasing lithic particles. These particles descend and amass at the base, creating sedimentary beds with an approximate porosity of 20% (Fig. 7a–c).

Our simulations further suggest that after ~ 150 Ka, this process of localized melting and phase segregation within the frozen strata results in the formation of multiple aquiferous chambers. These chambers typically measure around 1–2 km in width and 400–600 m in thickness, with these dimensions being dependent on the rate of intrusion heating (Fig. 7a). The distance between the centers of adjacent chambers is estimated to be around 3–4 km (Fig. 7a). By ~ 300 Ka, these chambers have expanded and merged, forming interconnected subsurface conduits (Fig. 7b,c). The dimensions and spacing of these conjoined chambers correspond to the amplitude of subsidence compartmentalization observed within the northwest region of Hydraotes Chaos, as exemplified by the elevation profile from point Y to Y′ depicted in Fig. 2.

Figure 7d,e illustrate the effects of magmatic heat injection at 1.0 W/m^2^ and 2.0 W/m^2^ levels, respectively. For the lower heating scenario, by ~ 150 Ka, the top of the liquid cavity reaches to about 1100 m below the surface (Fig. 7a,b). On the other hand, for the higher heating scenario, the top of the liquid cavity is observed at a shallower depth of approximately 780 m (Fig. 7c). Hence, accentuated subsidence might be a function of heat flow and not just the amount of buried water, suggesting that there could be vast swaths of thermally undisturbed subsurface mudstones retaining an elevated water ice content. Specifically, our simulations validate the geophysical feasibility of the proposed segregation mechanism at the scales observed within NW Hydraotes segmented floors, generally close to ~ 5 km cells (e.g., Fig. 2b).

## Discussion on the significance of Amazonian sedimentary eruptions in Hydraotes Chaos

### Gas-charged mudstones and evaporites as sedimentary volcanism sources

A critical observation is that while the floors of Hydraotes Chaos include widespread pitted cones and dome-like structures, these features do not occur on the chaotic terrain mesa surfaces (Fig. 5a). This distribution indicates an origin connected to the geologic composition of a stratigraphic zone underlying the mesa-forming materials. Such a morphogenetic connection is common in terrestrial sedimentary volcanism^74,75^.

In contrast, while intrusive magmatism can also generate pitted cones and dome-like features, its broad stress fields yield these extrusions without regard to the geological substrate types present^76–78^. Hence, we interpret that the pitted cones and dome-like structures are mud volcanoes and diapirs, respectively. Komatsu, et al.^79^’s and Brož, et al.^80^’s documentation of possible mud volcanism within other southern circum-Chryse locations supports this interpretation. Furthermore, the proposed mud volcanoes fall into Komatsu, et al.^79^’s Type 1 category (steep-sided cones typically with a summit crater, Figs. 2c, 5b).

On Earth’s sedimentary depocenters, mud volcanoes and mud diapirism coexist over breached gas-charged, muddy substrates^81,82^. Therefore, we suggest that an equivalent regional stratigraphy beneath Hydraotes Chaos, a detrital basin, could account for the proximal coexistence of these features. The existence of a clathrate stratigraphy has been previously postulated as a cause of the collapse leading to chaotic terrain formation^23,24,83^. In the context of our findings, we propose that the deeper parts of the buried, ice-rich mudstones could have also been clathrate bearing and that the prominent subfloor erosion leading to subsidence led to their unloading and decompression-triggered dissociation. In addition, heat released from the proposed magmatic intrusion (Fig. 7) could have also triggered or enhanced their dissociation.

While mud volcanoes and diapirs are typically situated within subsided floors (Fig. 5b–d), they display no signs of fracturing or deformation attributable to the extensional stresses incurred during the surface down-warping process. Given these observations, we propose that the emergence of these structures took place post the subsidence event. This chronological order implies that overpressure releases following crustal unloading could have triggered the buoyant rise of gas-charged muds, thereby instigating both mud volcanism and diapirism.

**Figure 8:**
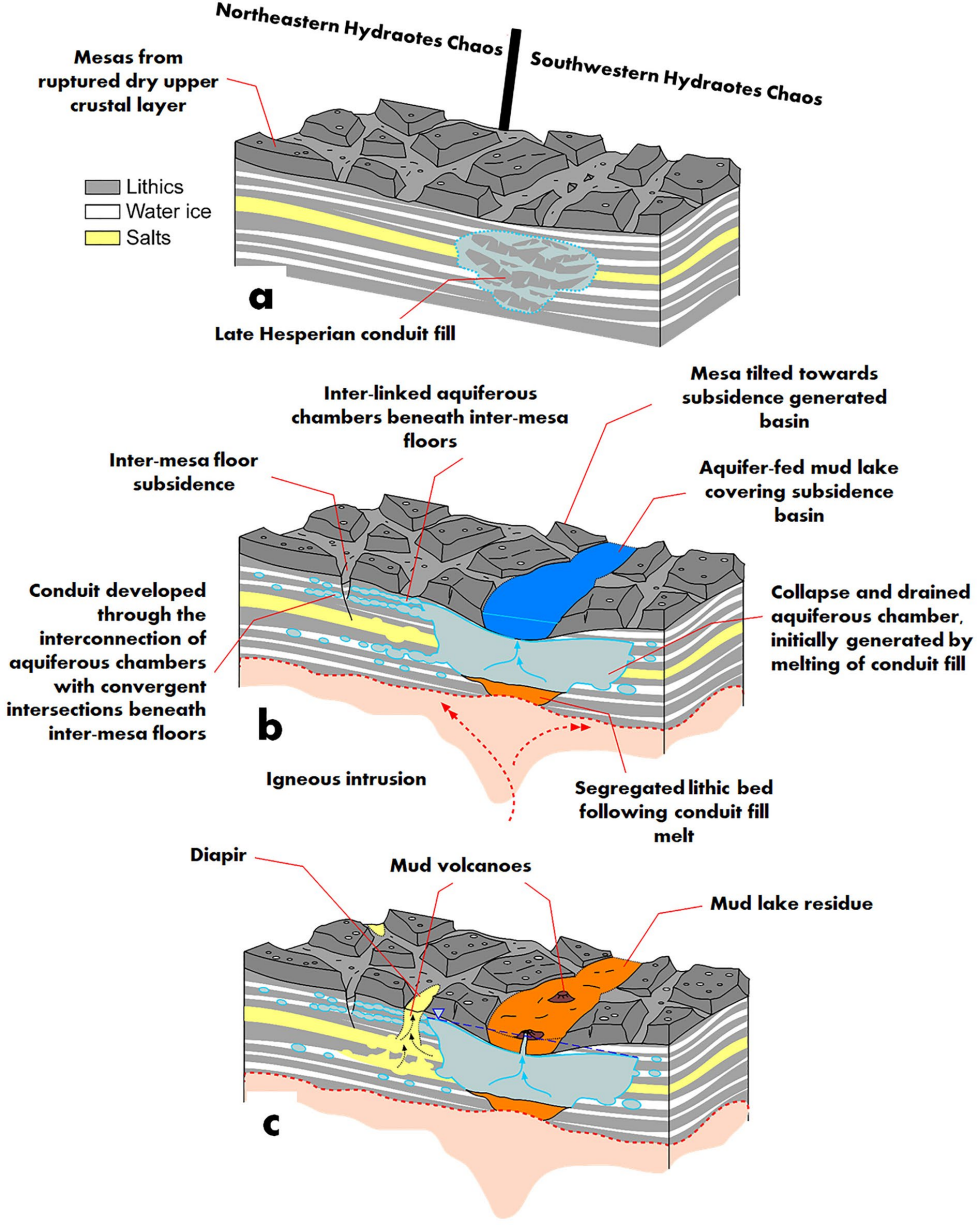
Stratigraphic and structural evolution of Hydraotes Chaos during Middle Amazonian. (**a**) Simplified stratigraphic depiction of Hydraotes Chaos during the Late Hesperian, providing a base for the subsequent, Amazonian hydrogeologic resurfacing history. (**b**) Illustration of the morphological changes driven by Middle Amazonian intrusive magmatism. Differentiation of aquiferous chambers from icy mudstone induces geological instabilities, propagating fractures to the inter-mesa floors, instigating subsidence, and stimulating the formation of sedimentary volcanic cones within NW Hydraotes. Concurrently, melting of a lowered Late Hesperian conduit fill within SE Hydraotes Chaos gives rise to a substantial aquiferous chamber featuring a segregated lithic base. Its massive scale precipitates an energetic collapse, initiating high-energy outflows that transport water sediments and fragmented ice into the resultant depression, thereby fostering a mud lake. (**c**) Following extensive subsidence and mud lake formation, desiccation occurs, sealing the remaining aquiferous chamber. Despite desiccation, an elevated water table persists, likely attributable to a freeze-resistant composition, and establishes a potentiometric surface that instigates mud volcanism over the now-dry lake's sedimentary plains. Furthermore, the loss of shallow rigidity, a consequence of the melting phase, permits density inversions that trigger diapiric ascensions in subsidence areas.

Additionally, diapir-prone geological contexts are frequently associated with Earth's rifted-margin domains, where segmental loading linked to extension and overburden sedimentation on exposed evaporite layers, nested within extension-induced fault-block structures, foster diapiric ascents^82,84^. Drawing a parallel, we notice certain diapirs along inter-mesa troughs where they coexist with mud volcanoes yet are conspicuously absent from the SHPD (Fig. 5a). Our hypothesis posits that, in this area, diapirism might have been a byproduct of localized loading on evaporite layers initiated by upper crust fragmentation, with upwelling taking place in non-loaded sectors. The proposed evaporite layers might have been stratigraphically interbedded with mud-rich layers, explaining their co-existence with possible mud volcanoes (Fig. 8).

#### Considering mud volcano alternatives

The interpretation of mud volcanic features on Mars remains challenging and alternative interpretations are sometimes hard to rule out. For example, while Meresse et al.^85^ identified the Hydraotes Chaos’ pitted cones as silicate cinder cones, the lack of corroborative a’a lava textures (Fig. 4) typically associated with magmatic volcanism challenges this hypothesis. Moreover, the study by Dapremont and Wray^86^ suggests that the absence of peripheral flows around Hephaestus Fossae's pitted cones is consistent with a magmatic origin.

In contrast, Hemmi and Miyamoto^87^, Hemmi and Miyamoto^88^, and Oehler and Allen^89  — ^ studying pitted cones in Terra Sirenum and Acidalia Planitia — noted low gradient flank slopes (< 10°), suggesting terrestrial mud volcanoes. This gradient diverges from those for cinder cones, which generally exhibit ~ 30° flanks^90^. Our local measurements in Hydraotes Chaos also show ~ 11° cone slopes (Fig. S4), favoring the mud volcano hypothesis.

However, we note that both lack of peripheral flows and steeper cone flank angles could imply wind erosion, as much as a magmatic origin. Such morphometric convergence complicates Martian volcano/mud volcano classification, underscoring the necessity for more in-depth studies on the influence of degradation processes on their morphology.

Hence, we do not discount the role of magmatic volcanism in forming some pitted cones. In fact, albeit infrequently associated with cinder cones, terrestrial mud volcanism can sometimes be linked to magmatic-induced geothermal activity^91^, resulting in the coexistence of mud and silicate volcanoes, such as in the Copper River Basin^92^.

In addition, while permafrost features like pingoes often coexist with volcanic elements, such as those in Bering Land Bridge National Park^79,93^, mud volcanoes are seldom associated with pingoes on Earth^94^. However, we acknowledge that a freezing mud lake in Hydraotes Chaos could have facilitated their formation^95,96^, potentially contributing to the regional pitted cones and domes.

Finally, although we also consider that it is also possible that some are eroded mountains, we note that these generally have more irregular shapes and should expose stratigraphy consistent with the surrounding mesas.

#### Probing evaporite-controlled diapirism and geological structure

Our observations reveal that the Chaos' eastern boundary is characterized by a pattern of ridges and troughs, notably elevated compared to the encompassing chaotic terrain, and running parallel to the proximate highland scarps (Fig. 5e). Possible diapirs are present in these troughs, yet no signs of evolved subsidence or mud volcanism are detected (Fig. 5f). Contrasting the more extensive low-lying areas (Fig. 2a,b), we interpret the absence of such subsidence as indicative of a lack of subsurface chambers beneath these floors.

We propose that the diapirs emanated from a deeper salt layer within the unexposed, extensively subsided chaotic terrain (Fig. 8), where highland-forming mudstones have undergone geothermally driven expulsions. These deeper layers may either extend from the evaporites in Valles Marineris, as suggested by Montgomery et al.^97^, or contribute to the broader Noachian marine sequence discussed by Rodriguez et al.^39^.

The presence of an expansive, ancient evaporite stratum beneath the mudstone unit could have served as a gas-impermeable lithological boundary^98–100^. This configuration sheds light on a potential gas trap mechanism. In response to pressure-induced fracturing of the overlying strata, this arrangement could have catalyzed sedimentary upwellings into the overlying mudstones, leading to mud volcanism throughout Hydraotes Chaos and probably other areas in southern circum-Chryse, such as documented by Komatsu et al.^79^ and Brož et al.^80^.

The heterogeneous rheology of the proposed stratified sequence, coupled with the higher density of the overlying mudstone materials relative to the evaporites, could have instigated diapirism^82,84^. Furthermore, the effect of extension^16,18,85^ on such stratigraphy could have initiated boudinage deformation, acting as a precursor to diapirism^101,102^. The boudinage process, which results in the creation of elongated rock structures through differential deformation, can facilitate the development of diverse dome fields. These types of formations are visible in Earth's salt domes^103^, glacial ice structures^104,105^, and permafrost features^104,106^, and might be present within the study region.

##### *Evaporite emergence from aquiferous chambers*

An alternative hypothesis to the ancient salt layer, which we explore here, is that some diapirs could have emerged from segregated salts within the aquiferous chambers. Although the mineralogy of the salts on Mars is not fully understood, we can still make predictions about their behavior based on our knowledge of salt diapirs, shale diapirs, and mud volcanism on Earth, as well as the properties of the materials found on Mars.

In-situ investigations have detected and characterized some Martian evaporite materials suggestive of surface and near surface water-rich settings at Meridiani Planum^107^, Gusev crater^107^, and Gale crater^108,109^. We postulate that, as these aqueous chambers underwent progressive cooling, a subsurface accumulation of brine salt precipitates and ice particulates could have occurred within them (e.g., for a suggestive study, see Travis et al.^110^).

We recognize the need in future work for detailed numerical modeling to account for the thermochemical conditions affecting precipitation and possible compositional shifts during diapiric ascension. However, current geochemical studies suggest probable precipitation of hydrated salts, including hydrohalite (NaCl·2H_2_O), gypsum (CaSO_4_·2H_2_O), kieserite (MgSO_4_·H_2_O), starkeyite (MgSO_4_·4H_2_O), epsomite (MgSO_4_·7H_2_O), meridianiite (MgSO_4_·11H_2_O), magnesium sulfate dodecahydrate (MgSO_4_·12H_2_O), and mirabilite (Na_2_SO_4_·10H_2_O)^83,108,111–113^. Additionally, hydrothermal hydroxylated salts such as jarosite (KFe_3_ (SO_4_)_2_ (OH)_6_) could have also been deposited^114^.

Upon formation, the evaporite lenses would have gained buoyancy, gradually ascending as solid-state or solid–liquid diapirs towards the surface, potentially breaching the subsided topography. For instance, the eutectic of magnesium sulfate and water occurs at approximately 269.25 K and 17 weight-% MgSO_4_ at low pressures, which then solidifies into a mixture of ice plus meridianiite (MgSO_4_·11H_2_O) or the dodecahydrate (MgSO_4_·12H_2_O). Under such conditions, the brine exhibits a density of about 1193 kg/m^3^, while the frozen mixture has a density of 1123 kg/m^3^^115^. It is noteworthy that meridianiite (MgSO_4_ undecahydrate) and the dodecahydrate phase have densities around 1510 kg m^−3^. Given these brine compositions, the density contrast with conventional rocks could have been sufficient to propel the upward movement of the diapir into the subsided valley floors, provided thermal conditions supported soft rheologies.

Subsequently, ice/salt diapirs originated from these strata and ascended to the surface (1–6 in Fig. S5). Given the heating and dehydration of these salt layers and diapirs, they could have transitioned into regional sources of fluid eruptions and surface mud lakes (7, 8 in Fig. S5).

### Unraveling the link between subsurface conduit networks and regional hydrology patterns

We postulate that the aqueous chambers may have interconnected to establish a network of subsurface conduits. In Figure 7, liquid brine is modeled at a depth of ~1200 m, where hydraulic pressure equilibrium with the overlying rock would cause a pressure of ~12.9 MPa, assuming a crust density of 2900 kg/m³. This pressure is calculated using the formula P = 2900 kg/m³ × 3.72076 m/s² (Mars gravitational acceleration) × 1200 m.The compressibility data of magnesium sulfate solution, as delineated by Hogenboom, et al.^115^ (see, for example, their figure 15), suggests that cryogenic conditions could have fostered the development of much greater hydraulic pressures within these conduits. However, the geology suggests that confining materials, possibly akin to shale, must be weak and unable to bear such high stresses, with shale's tensile strength at ~5 MPa^148^. Hence, the cumulative weight of overlying strata, and the expansion of cryo-trapped ice within these formations could have induced significant overpressures. Sustaining such significant pressure would be unlikely without several episodic release events. Besides causing deformation in the chamber walls, these overpressure-induced eruptions could have promoted the progressive expulsion of residual non-frozen mud from the conduits. This process could have initiated eruptive events and mechanical instabilities leading to local tensional and compressional features, ultimately causing the subsidence and collapse of the conduits.

In Hydraotes Chaos's northwestern region, geologic subsidence predominantly affected inter-mesa areas. Despite a lack of substantial compensatory sedimentation, scattered mud volcanoes and diapirs, which seem to have formed post-subsidence, are evident. Conversely, the southeastern region underwent extensive subsidence, leading to a prominent basin offset by aquiferous outbursts that facilitated the formation of a lacustrine mud body, of which the SHPD is a residue.

We posit a model linking the hypothesized conduit networks with the emergence of mud-rich water in Hydraotes Chaos's southeastern region. Our model suggests the formation of a broad subterranean conduit network in the northwestern area, converging into a significant aquiferous chamber in the southeast (Fig. 8b). The chamber's size-induced instability instigated the localized expulsion of mud-rich water, initiating the formation of the proposed mud lake (Fig. 8c).

Furthermore, we suggest that the sustained flow from northwestern conduits into the collapsing chamber extended the duration of groundwater outflows. This increased the available groundwater volume for lake generation and possibly supplied a mechanism for pressurization following the freezing of its surface.

In addition, subsequent to the paleolake's disappearance, these subsurface conduits might have temporarily maintained an elevated water table, presumably composed of freeze-resistant brines. This water table could have established a potentiometric surface, which in turn, might have also instigated mud volcanism over the floor of the now desiccated paleolake (Fig. 8c).

The large dimensions of the proposed aquiferous chamber beneath the southeastern Hydraotes Chaos implies that this region was juxtaposed over a subterranean zone marked by anomalously high ground ice concentration. The subsided basin in southeastern Hydraotes Chaos aligns with an adjoining highland's curvilinear depression. This depression, previously inferred as a Late Hesperian subsidence locale, is part of a broader highland subsidence system over proposed catastrophic-flood-feeding conduits^20^. Hence, we hypothesize that large volumes of ice amalgamated with mudstone fragments composing the conduit fill (likely as ice intermixed with siliclastic sediment^116^) were regionally sustained until the Middle Amazonian epoch.

#### Emergent diapiric patterns as an alternative control mechanism for the origin of subsurface conduit networks

Our numerically coupled model posits localized mudstone melting, induced by magmatic heat, as a potential catalyst for the formation of aqueous chambers (Fig. 8). Additionally, we hypothesize that these aquiferous chambers could have resulted from the heating of pre-subsidence diapirs that ascended into the proximate subsurface layer of the trough subfloors (Fig. 5a,e,f). Their hydrated salt could have facilitated the confinement of water within discrete subfloor volumes, thereby potentially enabling the geothermal generation of aquiferous chambers (as proposed by Travis, et al.^110^). Following their intersection, these networks would have served as precursors to tectonically guided, regional subsidence. Notably, recent research attributes some Martian depressions to collapse over devolatilized diapirs^93^.

### Furthering our understanding of aquifer stability in southern circum-chryse

The connection of outflow channels to the northern paleo-ocean indicates an enormous early Mars hydrosphere, which experienced significant drainage to the northern plains’ surfaces ~ 3.4 Ga (Late Hesperian^2,9,117^ (Tanaka et al.^6^)).

Our results indicate that ~ 1.1 Ga the hydrosphere generated a second paleolake within Hydraotes Chaos (the first one is thought to have formed during the Late Hesperian^45,46^), implying regional aquifers' endurance far beyond their Late Hesperian depletion event. Yet, the absence of overflow within Hydraotes Chaos suggests smaller discharge volumes than for the Late Hesperian counterparts, indicating possible shrinkage of the aquifer or its recharge capacity^15^.

Furthermore, our findings also suggest a history of Amazonian catastrophic flooding events, wherein both the source and deposition zones were confined within the circum-Chryse outflow channels. This finding further underscores the significance of Martian outflow channels as terminal receivers during the younger instances of substantial flooding.

Lastly, the considerable ~ 2.3 Ga time interval between the Late Hesperian subsurface-conduit-fed catastrophic floods (~ 3.4 Ga) and the Middle Amazonian (~ 1.1 Ga) paleolake occurrence within Hydraotes Chaos suggests that the proposed, subsidence-lowered conduit section beneath southwestern Hydraotes Chaos must have endured over multiple billion years (significantly outlasting any terrestrial karstic-fluvial systems). Consequently, we postulate that this conduit section (and most likely others) had a mechanically resilient structural framework, capable of withstanding the erosive effects of the intense, Late Hesperian groundwater flow and hydrostatic pressure variations.

### Multi-billion year biosignature preservation, and concentration through aqueous releases

Our proposed ice-rich mudstone stratigraphy subjacent to Hydraotes Chaos potentially developed during large-scale marine sedimentation, leading to the formation of the highland of southern circum-Chryse during the Middle Noachian (~ 3.94 Ga to ~ 3.83 Ga)^6^. This subsurface stratigraphy presents an opportunity for post-Noachian habitability and radiation-shielded preservation of potential molecular biosignatures.

Our simulations suggest potential habitability in aquiferous chambers from ice-melting in mudstones. Different heating rates (Fig. 7d,e (1 W/m^2^), 7f. (2 W/m^2^)) create extensive 0–100 °C domains (Fig. S6), bolstering chances for biosignature preservation, and providing windows of potential habitability that could have lasted a few hundred thousand years.

The proposed Late Hesperian and Middle Amazonian Hydraotes Chaos paleolake formations represent two distinct drainage events from these potentially habitable water-rich environments and showcase an argument for Martian aquifers regionally persisting for billions of years. This possibility is supported by evidence of billion-year-scale groundwater retention on Earth^118^.

Our hypothesis is that a water-lithic phase segregation process generated an aquiferous chamber within the mudstone stratigraphy. The groundwater within the chamber could have acquired diverse biosignatures previously dispersed within the distinct layers forming the Noachian mudstone stratigraphy. The chamber collapse would have released these biosignatures, along with aquifer sediments and water, ponding to form the proposed mud lake. As the lake gradually dissipated, possible biomolecules could have been precipitated and concentrated within its sedimentary residue (the SHPD). Furthermore, while the SHPD is ~ 1.1 Ga, the pitted cones that superpose it could be younger, perhaps offering sedimentary deposits that were even more recently extruded, and hence, less exposed to the bio-damaging surface environments of Mars.

### Landing site considerations

#### Preliminary landing target selections

Our proposed geologic scenario encompasses interlinked intrusive magmatism, voluminous groundwater melt generation and evacuation, followed by gas-charged mud decompression and eruptions (Fig. 8). We posit that the investigated pitted cones and dome-like features within Hydraotes Chaos floors (Fig. 5) largely represent mud volcanoes and diapirs, respectively. Furthermore, the morphological traits of these features can guide us to distinct outcrops of mud volcano deposits and diapiric extrusions. Analyzing these samples can elucidate formation conditions, geochemical interactions, and potentially reveal isotopic signatures of ancient environments. Residual brine precipitates and potential biosignatures may offer insights into their original composition and help us interpret the historic hydro-climatic factors influencing these structures' evolution.

Our selection of potential mud volcanoes focuses on pitted cones bearing surrounding deposits fringed by irregular scarps (red arrows in Fig. 5b). This pattern aligns with water-charged mud breccia eruptions common to sedimentary volcanism (e.g., Fig. S7a). As for the selection of potential diapirs, we are particularly interested in domical structures with elevated fractured surfaces (blue arrow in Fig. 5c, Fig. S7b) and inward-tilting unconformities surrounding concentric layers (blue arrow in Fig. 5d, Fig. S7c), traits consistent with emerging and eroded diapirs, respectively^119,120^. Also, some domes are topped by sloping mesas extending to plains with distinctive lobate edges (Fig. 6), suggesting that gas-infused diapirs may have surfaced as muddy upsurges. Regional diapir summit knobs (Fig. 5d) could be sediment-made venting structures for mud and gas^79,93^.

#### Rationales for high biomarker preservation within the SHPD

We hypothesize that the SHPD, originating from a subsurface, Noachian stratum, could be a trove of preserved molecular biosignatures. Hence, we advocate for its prioritization as a prime target for astrobiological investigation.

Our investigation indicates that the age of SHPD emplacement, around 1.1 Ga, corresponds with Earth-based timelines for the preservation of core structures of lipid biomarkers, suggesting it could potentially be a repository of preserved biosignatures^121–123^. Moreover, numerous environmental factors at SHPD could have amplified biomarker preservation. Particularly, the cryogenic, desiccating Martian conditions, along with the lack of metamorphic activity, might have contributed to biomarker longevity^124^. Corresponding terrestrial environments, which foster active ecosystems, are well-documented to maintain biomarker integrity over substantial geologic timescales^125–128^.

However, we should note that organic matter residing within the upper 2–3 m of the Martian surface is subject to degradation from ionizing radiation, primarily solar energetic particles (SEPs) and galactic cosmic rays (GCRs). Degradation mechanisms include direct impacts that cleave bonds, or indirect effects arising from interactions with secondary gamma rays, oxidants, and radicals produced by the incoming charged particles^129,130^.

Experimental and model-based projections suggest that organic molecules at depths of 0–10 cm will undergo degradation based on their mass. Molecules with atomic mass units (amu) less than 100 are expected to degrade within a timeframe of 1 Ga, whereas those exceeding 300 amu are projected to degrade within 0.3 Ga. However, within the depth range of 10 cm to 3 m, the overlying material provides sufficient shielding to significantly extend these molecular lifetimes^129,131,132^.

Several factors, including mineral-organic interactions and desiccation, can slow the degradation process^130,133^. The radiation exposure of sedimentary strata, from charged particles and secondary gamma radiation, is primarily confined to the topmost 3 m. Hence, organics present below this threshold are anticipated to be effectively shielded from radiation degradation^129,131,134^.

In the SHPD, possible ongoing wind erosion might expose preserved organics faster than radiolytic degradation could alter them, thus improving the detectability of ancient organics. This elevates the site as a unique and highly promising target for astrobiological missions seeking Martian life's molecular traces. We propose that in-situ sampling of the upper stratigraphy, using a rover or stationary lander^135^, would further increase the chances of reaching organic-rich soil beds.

Our suggested location, a desiccated mud lake, may contain concentrated surface and near surface, ancient organics. The SHPD is likely in contrast to many other Martian sites, which lack such geologic history involving biosignature concentrating mechanisms. Some experts advocate geological sample return, rather than in-situ analysis, as the most effective approach for detecting Martian biosignatures, due to the potential for concentrations to fall below the detection threshold of existing in situ sensing technology^136^. This concern relates to the types of instrumentation previously deployed, considering their limitations in spatial resolution, sample size, and analytical instrument limits of detection^137–139^. Consequently, future astrobiological missions should adhere to rigorous extraction protocols, drawing from terrestrial methodologies adapted for spaceflight conditions during sample return. These must consider the possible variations in organic dispersion by securing sizeable soil samples (~ 10 s of grams), utilizing low-temperature solvent extraction, eliminating inorganics, concentrating organics, and having a low-limit of detection analyzer^140^.

## Broader implications and significance

### Reevaluating our understanding of the Martian buried hydrosphere through southern Circum-Chryse highland stratigraphic reconstructions

Previous studies indicate that the mesas in Hydraotes Chaos, and others scattered across the southern circum-Chryse region's chaotic terrains, are remnants of rifted highland areas that experienced significant subsidence during the Late Hesperian^16,18,39,85,141^.

The mesa scarps preserve polygonal outlines, relics of the aforementioned subsidence phase (Fig. 8a). This retention pattern implies that these mesas are composed of relatively resistant materials, and notably, these materials likely did not contain substantial volumes of water ice, which could have led to periglacial and glacial modification of the scarps, as believed to have occurred in the Deuteronilus Mensae region of northern Arabia Terra^142^.

The character of combined sedimentary volcanism and diapirism within Hydraotes Chaos suggests an underlying stratigraphy made up of a buried, ice-rich, gas-charged mudstone and an underlying evaporite layer (Fig. 8). In addition, our model postulates that this stratigraphic assembly underwent substantial sedimentary eruptive evacuation and extensive diapiric deformation during the Middle Amazonian (Fig. 8). As a result, the subfloor of Hydraotes Chaos may be highly heterogeneous, possibly featuring extensive overturned strata.

Our revised upper crustal stratigraphy places the southern circum-Chryse aquifers within a gas-laden, ice and salt-rich mudstone layer, overlying an evaporite stratum. This vision departs from Clifford's model by suggesting that, at least in this region, the confinement of groundwater was not due to an ice-saturated cryosphere but was influenced by the low permeability of mudstone/evaporites. In addition, our regional model stands in contrast to the global sedimentary hydrosphere proposed by Scheller et al.^30^, proposing that the subsurface water released during chaotic terrain collapse existed as ice and/or evaporite layers, rather than being bound within clay minerals.

#### Late Hesperian marine hydraulic equilibrium

Previous research indicates a hydraulic balance between an inland sea in southern circum-Chryse and the northern ocean, potentially maintained through permeable structures in Hydraotes Chaos^45^. If the proposed Hydraotes Chaos-underlying mudstones in this region extended far northward into the northern lowlands, partial ice melt could have created networks of water-filled conduits, sustaining this equilibrium. Additionally, or alternatively, this interconnection may have occurred within an underlying stratified evaporite layer.

### Subsurface gas traps in Hydraotes Chaos: a hypothesis for hydrogen depletion and the existence of deep aquifer-bearing strata

The stratigraphic configuration in Hydraotes Chaos, characterized by mudstones superimposed on evaporites, mirrors terrestrial mega gas fields. On Earth, argillaceous materials and evaporites commonly serve as caprock materials for gas fields, gypsum salt rock exhibiting the most effective sealing performance^98^. We propose that the mudstone's impermeability could have facilitated the sequestration of volcanic effluents, including carbon dioxide and methane, potentially catalyzing clathrate genesis.

Later, magmatic intrusions or other pressure/thermal disturbances could have triggered clathrate dissociation and subsequent outgassing, potentially causing sedimentary volcanism. This process could also have created sealed gas pockets, which might have undergone repeated cycles of confined sublimation and condensation. Furthermore, we propose that the mechanism of volatile entrapment within the clay-rich and evaporitic stratigraphy could explain the scarcity of hydrogen (~ 1.8 WEH, Fig. 3B)^143^ in Hydraotes Chaos' regolith, attributed to low diffusion rates.

## Conclusions and summary

The Martian chaotic terrains and outflow channels, subjects of study since the 1970s, are believed to signify substantial subterranean water sources. However, the identification of aquifer-released fluids in these channels presents a challenge due to the interference of eroded highland materials and, in higher latitudes, recent ice and aeolian mantles. In this investigation, we document a sedimentary deposit within the Hydraotes Chaos region and posit its formation as a result of Middle Amazonian groundwater and mud, released from an aquifer mostly underlying a deep basin within the chaotic terrain. We propose that the discharges into the basin rapidly, and without channelized flow, ponded into a lake  and that its sedimentary residue forms an extensive deposit consisting of aquifer-released materials. 

We hypothesize that the sedimentary eruption could have been instigated by geothermal heat acting upon ancient, gas-charged mudstone deposits abundant in water-ice strata. This thermogenic activity would have segregated the water from the sediment, facilitating the formation of a series of interconnected chambers filled with water, or aquiferous chambers. With time, these chambers experienced structural failure, unleashing muddy water discharges, leading to widespread subsidence throughout the area, and consequently giving rise to a lake in the southeastern part of Hydraotes Chaos.

Following the drainage, subsidence came to a halt. However, we propose that the consequential unloading resulted in decompression of the gas-charged mudstone unit, instigating mud volcanic eruptions and ascension of diapirs into the subsided floors. Additionally, we propose that some of these diapirs could have emerged from a deeper evaporite layer, which could span a supra-regional extent.

Our age estimations of the mud lake point towards its formation around ~ 1.1 Ga, which is approximately ~ 2.3 Ga post the peak of outflow channel activity in the region. This time correlation implies that aquifers on Mars might have persisted for billions of years, potentially preserving habitability and stable biosignatures for significantly more extended periods than other water-related materials that were deposited at the surface earlier in the planet's history.

Our investigations suggest that the southern circum-Chryse region, apart from exhibiting collapsed terrains and groundwater release landscapes typical of chaotic terrains and outflow channels, also contains aquifer-released materials. These materials were likely sourced from a stratigraphy consisting of clay and evaporite deposits located beneath a dry, resilient layer, which subsequently disintegrated, culminating in the formation of the mesas observed in the chaotic terrain.Our investigation does not show evidence indicative of the long-held view of a regional hydrosphere confined and pressurized beneath an ice-saturated cryosphere^9^.

## Directions of future work

In this article, we document details on how Amazonian hydrogeology operated within the southern circum-Chryse region of Mars. Most of the region’s previous work focuses on the occurrence of outflow channel forming, ocean-sourcing, catastrophic floods released from aquifers during the Late Hesperian^2,6^. However, some other investigations reveal that the regional subsurface also underwent catastrophic floods episodically during the Amazonian Period^144,145^, which has lasted ~ 3 Ga, essentially most of the Martian history. Our findings suggest that intrusive magmatism regionally reactivated a residual aquifer situated beneath Hydraotes Chaos, releasing mud flows, which ponded and covered the chaotic terrain’s lowest zones. The resulting sedimentary unit offers an outstanding site for seeking biosignatures released from the upper crustal aquifers. Some important questions remain, and these should be explored through further research. Addressing them would concern, for example, determining the connection of the Amazonian floods to Mars ponding history, in particular discerning whether the floods fed into the northern plains to form younger seas as suggested by Fairén et al.^36^. A detailed, focused mapping effort to determine the distribution of Amazonian groundwater sources and water-laid sediments would be important for increasing the targeting repertoire of aquifer-released sediments for astrobiological considerations. Furthermore, the increased documentation would provide us with more information regarding the nature and diversity of the groundwater release mechanisms and the structural integrity and interconnectivity of the sources. For example, evidence of extensive catastrophic flooding during the Amazonian would be consistent with the development of conditions favorable to broad-scale hydrology, perhaps connected to warm paleoclimatic periods^144,145^. On the other hand, if the floods were localized, their trigger was likely an endogenic process such as intrusive magmatism^85,141,146^.

### Supplementary Information

Supplementary Information.

## Data Availability

The datasets used and analyzed during research are released and publicly available from NASA.
