# Author Correction: Exploring the evidence of Middle Amazonian aquifer sedimentary outburst residues in a Martian chaotic terrain

**DOI:** 10.1038/s41598-024-63710-8

**Published:** 2024-06-04

**Authors:** J. Alexis P. Rodriguez, Mary Beth Wilhelm, Bryan Travis, Jeffrey S. Kargel, Mario Zarroca, Daniel C. Berman, Jacob Cohen, Victor Baker, Anthony Lopez, Denise Buckner

**Affiliations:** 1 Planetary Science Institute, 1700 East Fort Lowell Road, Suite 106, Tucson, AZ 85719-2395 USA; 2 External Geodynamics and Hydrogeology Group, Department of Geology, Autonomous University of Barcelona, Bellaterra, 08193 Barcelona, Spain; 3 NASA Ames Research Center, Moffett Field, CA 94035 USA; 4 Department of Hydrology and Atmospheric Sciences, University of Arizona, Tucson, AZ 85721 USA; 5 Blue Marble Space Institute of Science, Seattle, WA 98104 USA; 6 University of Florida, Gainesville, FL 32611 USA

Correction to: *Scientific Reports* 10.1038/s41598-023-39060-2, published online 18 October 2023

The original version of this Article contained an error in the Abstract.

“Furthermore, the lake’s residue’s estimated age is ~1.1 Ga (~3.2 Ga post-peak aquifer drainage during the Late Hesperian), enhancing the prospects for organic matter preservation.”

now reads:

“Furthermore, the lake’s residue’s estimated age is ~1.1 Ga (~2.3 Ga post-peak aquifer drainage during the Late Hesperian), enhancing the prospects for organic matter preservation.”

The original Article has been corrected.

