# Supplementary material for: Exploring the evidence of Middle Amazonian aquifer sedimentary outburst residues in a Martian chaotic terrain
Source: Sci Rep. 2023 Oct 18;13:17524. doi: 10.1038/s41598-023-39060-2 (PMC10584912; doi:10.1038/s41598-023-39060-2)
Supplement: Supplementary file 1 — Supplementary Information. [file 41598_2023_39060_MOESM1_ESM.docx]

**Supplementary File to:**

**Exploring the Evidence of Middle Amazonian Aquifer Sedimentary Outburst Residues in a Martian Chaotic Terrain**

J. Alexis P. Rodriguez^1^*, Mary Beth Wilhelm^2^, Bryan Travis^1^, Jeffrey S. Kargel^1^, Mario Zarroca^3^, Daniel C. Berman^1^, Jacob Cohen^2^, Victor Baker^4^, Anthony Lopez^3^, Denise Buckner^1,5,6^

*^1^Planetary Science Institute, 1700 East Fort Lowell Road, Suite 106, Tucson, AZ 85719-2395, U.S.A.*

*^2^NASA Ames Research Center, Moffett Field, CA 94035, U.S.A.*

*^3^External Geodynamics and Hydrogeology Group, Department of Geology, Autonomous University of Barcelona, 08193 Bellaterra, Barcelona, Spain.*

*^4^Department of Hydrology & Atmospheric Sciences, University of Arizona, Tucson, AZ 85721, U.S.A.*

*^5^Blue Marble Space Institute of Science, Seattle, WA, 98104, U.S.A.*

*^6^University of Florida, Gainesville, FL 32611, U.S.A.*

Corresponding author: Alexis Rodriguez (alexis@psi.edu)

**Supplementary Figures
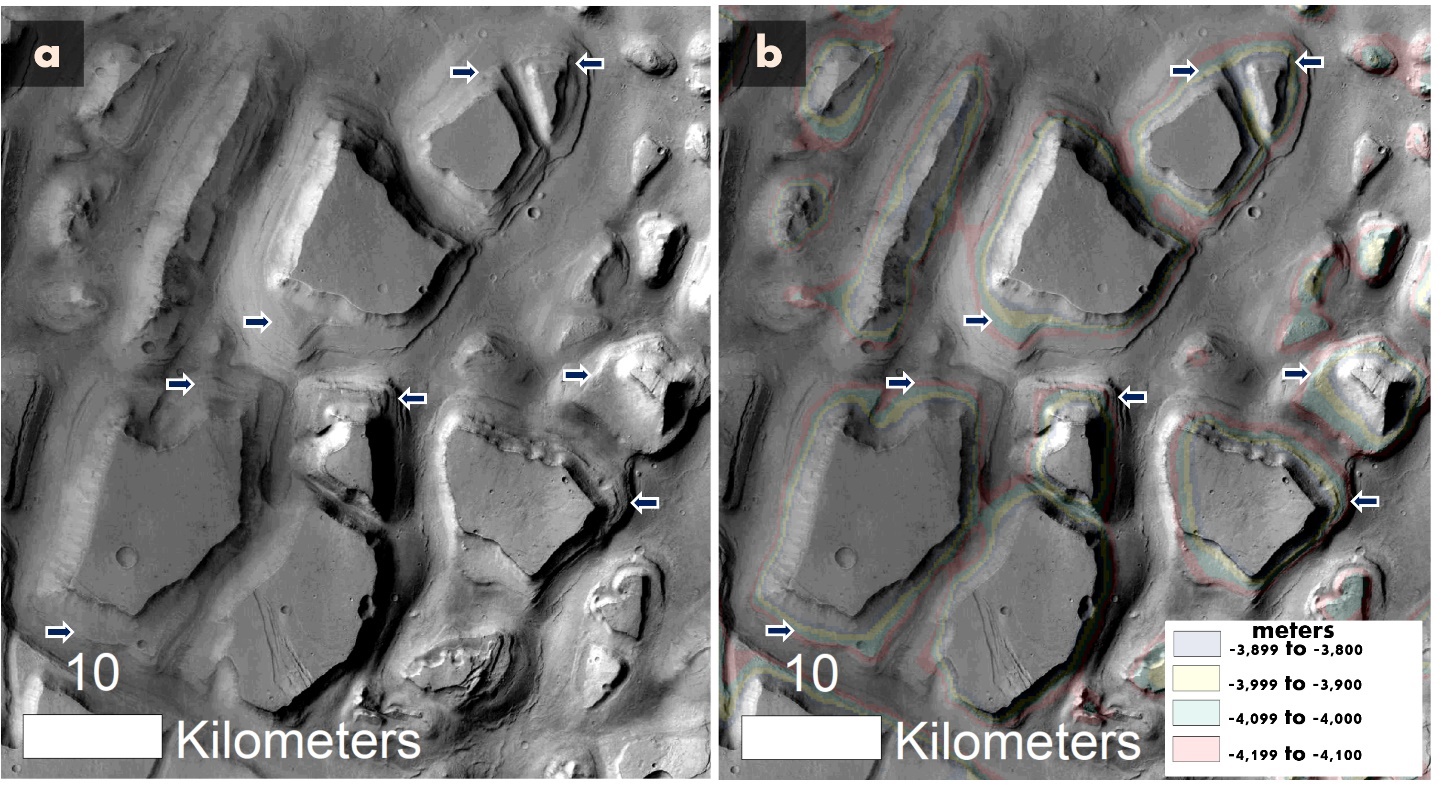
**

**Fig. S1 (a)** This view illustrates terraced mesas in the northwestern Hydraotes Chaos, previously interpreted as shoreline features. Notably, these terraces (marked by black arrows) extensively flank mesas of various dimensions. **(b)** This image features a semi-transparent DEM layer indicating that these terraces typically occur at elevations between -3,800 m and -4,200 m.

**(a)** This is a THEMIS daytime Infrared (IR) global layer of Mars (Resolution: 100 m/pixel. Data credit: Christensen, et al. ^1^); available at <http://www.mars.asu.edu/data/>). **(b)** This image features a High Resolution Stereo Camera (HRSC) - Mars Orbiter Laser Altimeter (MOLA) Digital Elevation Model (DEM) (Resolution: 200 m/pixel; data credit: MOLA data – NASA, HRSC data – ESA/DLR/FU Berlin), overlaid on a THEMIS daytime IR global layer (Resolution: 100 m/pixel; data credit: Christensen, et al. ^1^); available at <http://www.mars.asu.edu/data/>). Both figures were produced using Esri's ArcGIS 10.3 software (<http://www.esri.com/software/arcgis>).


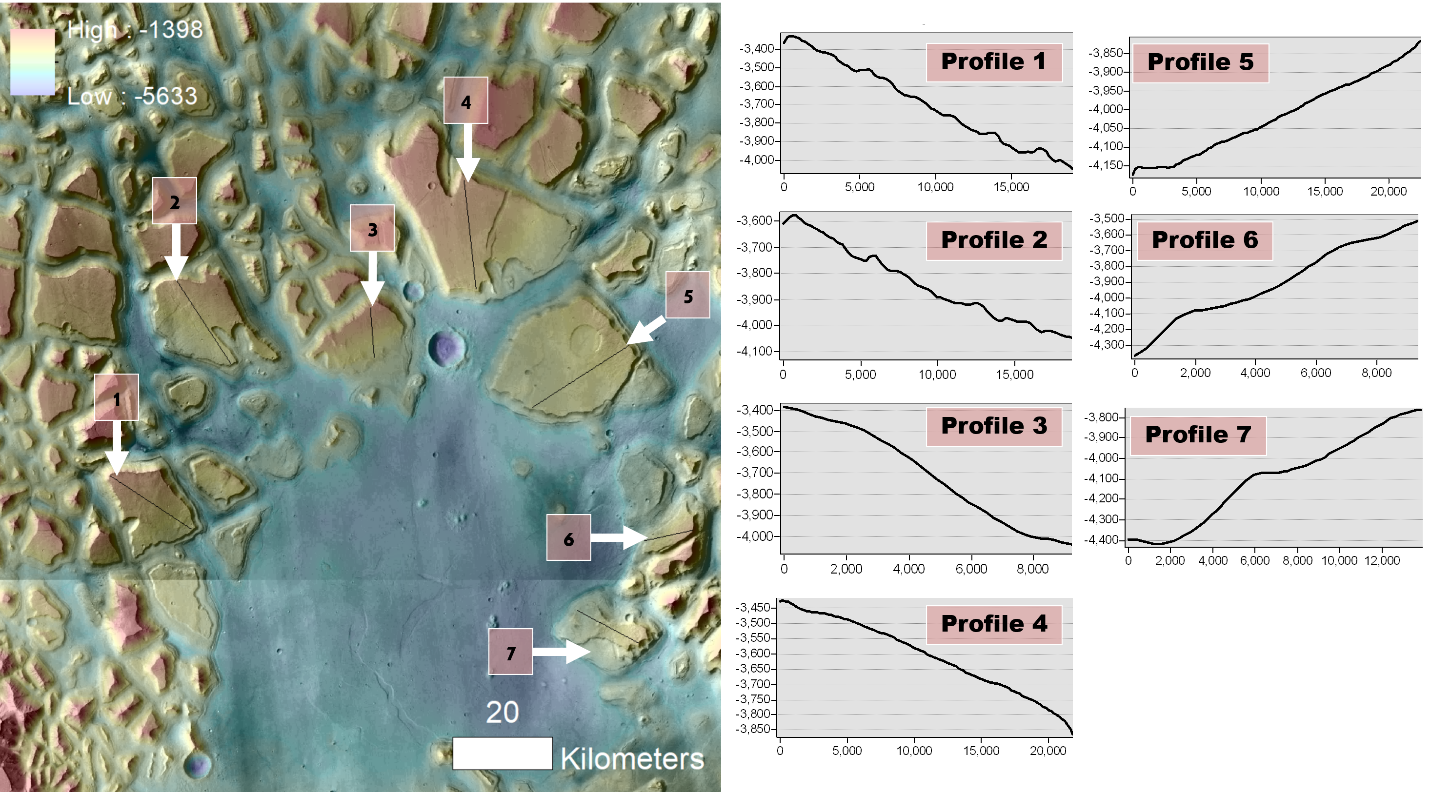


**Fig. S2** This view depicts the seven mesas identified in Figure 3a, emphasizing their tilt component oriented towards the Southern Hydraotes Plains Deposit (SHPD). The image is based on an HRSC-MOLA DEM (200 m/pixel, credit: MOLA data – NASA, HRSC data – ESA/DLR/FU Berlin) overlaid on a part of a CTX mosaic ^2^ with a resolution of 6 m/pixel (credit: NASA/JPL/Malin Space Science Systems; available at <https://www.msss.com/mro/marci/images/tips/mediatips.html>).

**
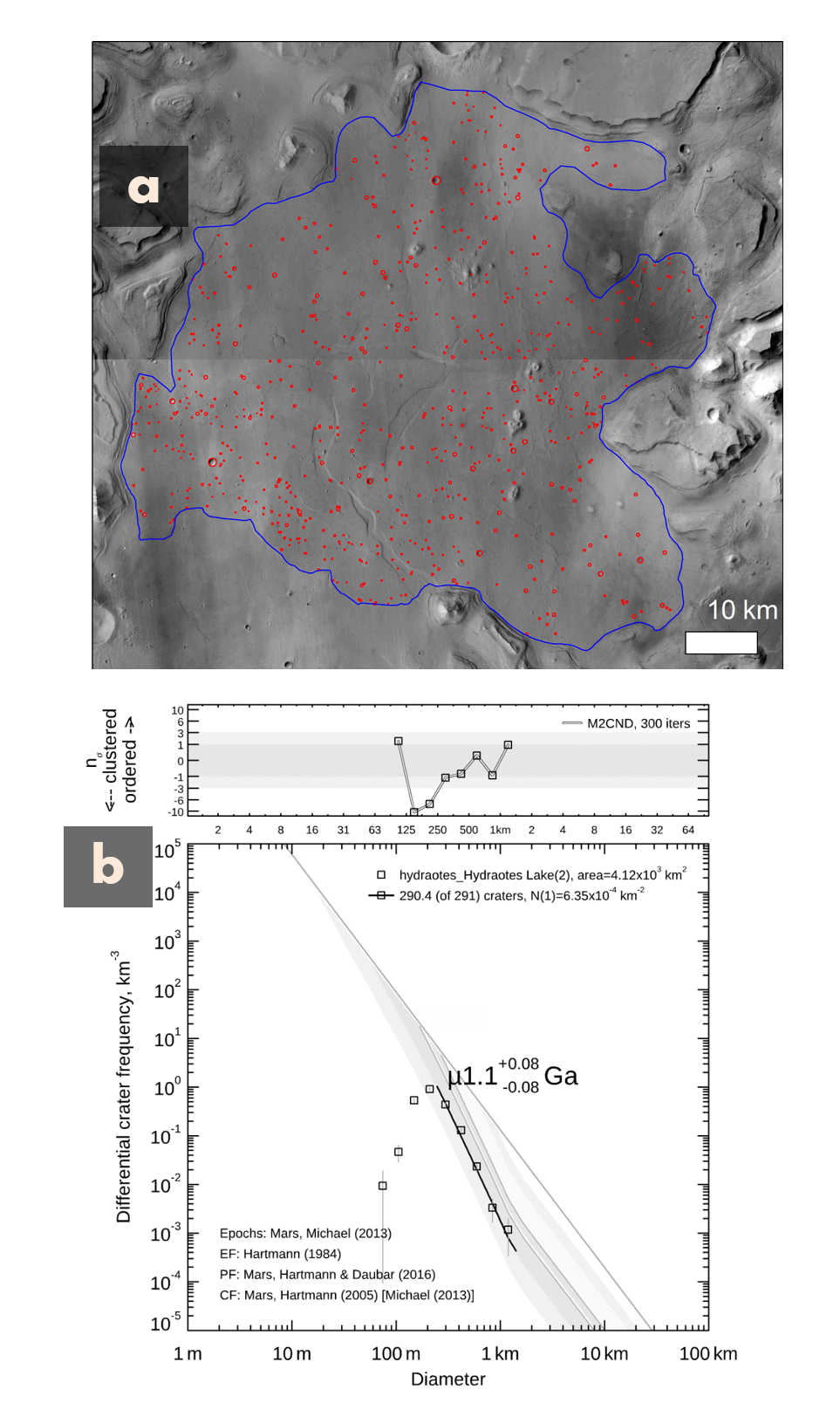
**

**Figure S3** **(a)** This image represents crater counts (marked by red circles) within the SHPD (outlined by a dark blue line). The base layer is a THEMIS daytime IR global layer (<http://www.mars.asu.edu/data/>, 100 m/pixel, credit: Christensen, et al. ^1^). **(b)** This chart presents a differential crater size-frequency distribution for the area of the Hydraotes Chaos floor outlined in panel (a). This distribution was calculated using the chronology function from Hartmann ^3^ as adapted by Michael ^4^ and the production function from Hartmann and Daubar ^5^. The model age was determined using a bin fit range from 250 m to 1.4 km.


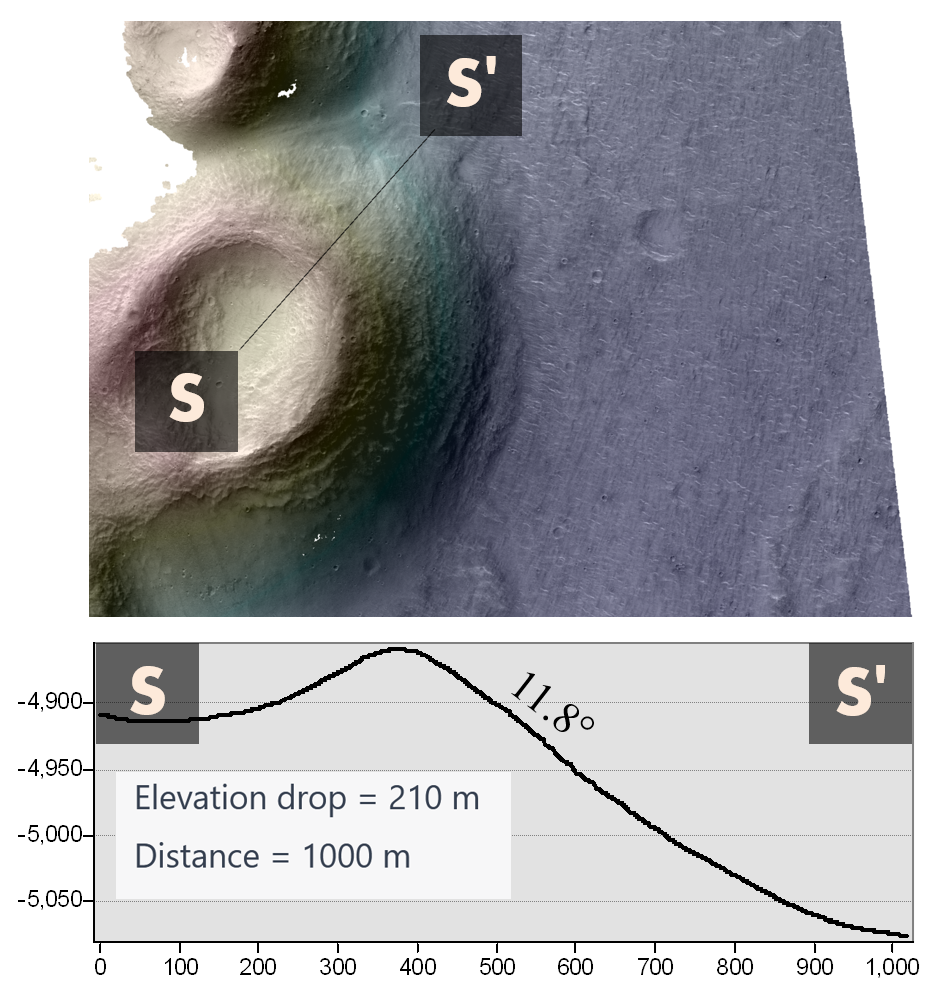


**Figure S4** A high-resolution perspective of a portion of a proposed mud volcano cluster, taken with the HiRISE DEM (source stereopair: ESP_025493_1800 & ESP_017634_1800), superimposed on a HiRISE image over a segment of ESP_025493_1800 (25 cm/pixel). This detailed viewpoint is centered at 0° 8'28.26"S, 33°41'56.46"W. Image credit: NASA/JPL/University of Arizona (<https://www.uahirise.org/media/usage.php>).


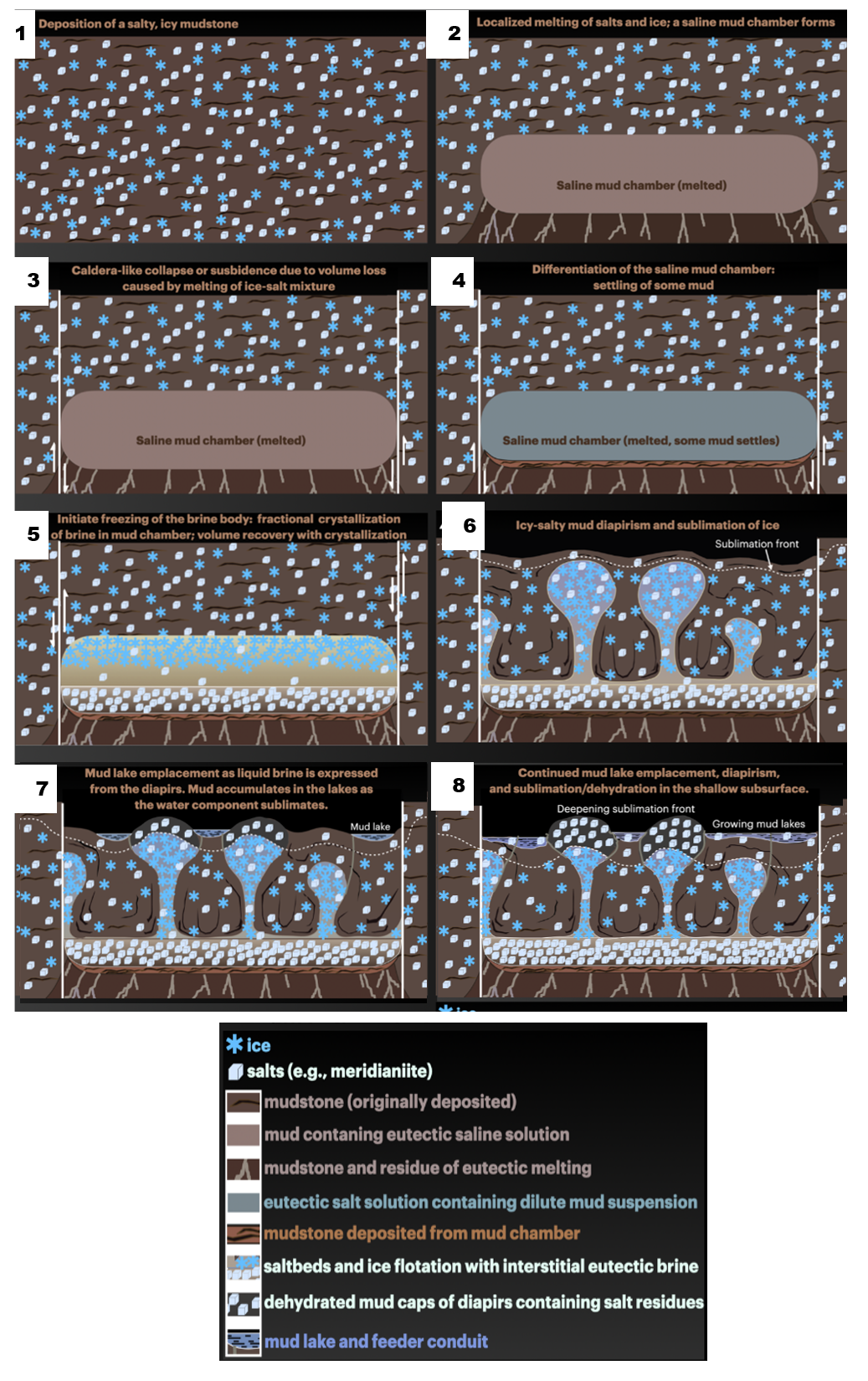


**Figure S5** Depicted here is a conceptual sketch, showcasing a variant of our model featuring water-filled aquiferous chambers. This model vividly illustrates the process of fractional crystallization leading to the formation of evaporite layers, from which ice/salt diapirs ascend to the surface (Stages 1-6). Upon dehydration, these diapirs transform into regional sources for surface mud lakes and contribute to the creation of depressions in areas subjected to diapir erosion (Stages 7, 8).


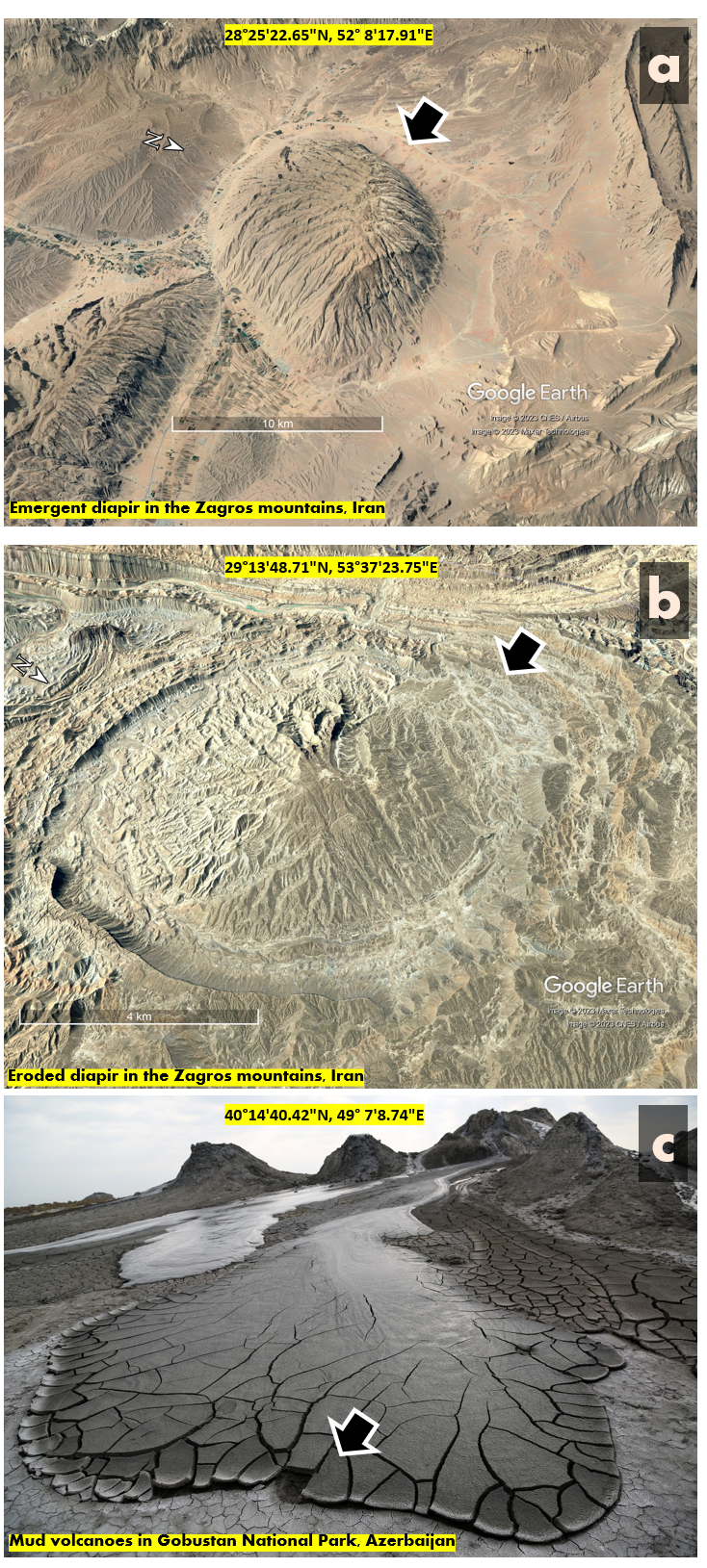


**Figure S6** **Panels (a) and (b)** depict diapirs (highlighted by black arrows) from the Zagros mountains of Iran, with panel (a) demonstrating initial uplift and doming, and panel (b) revealing the aftermath of erosion which exposes the concentric stratigraphy within the diapir. Panel (c) presents a desiccated mud flow breccia, illustrating fracturing and breakup. These images, courtesy of Y Nakanishi, are shared via Flickr under this license: <https://creativecommons.org/licenses/by-nc-nd/4.0/>.


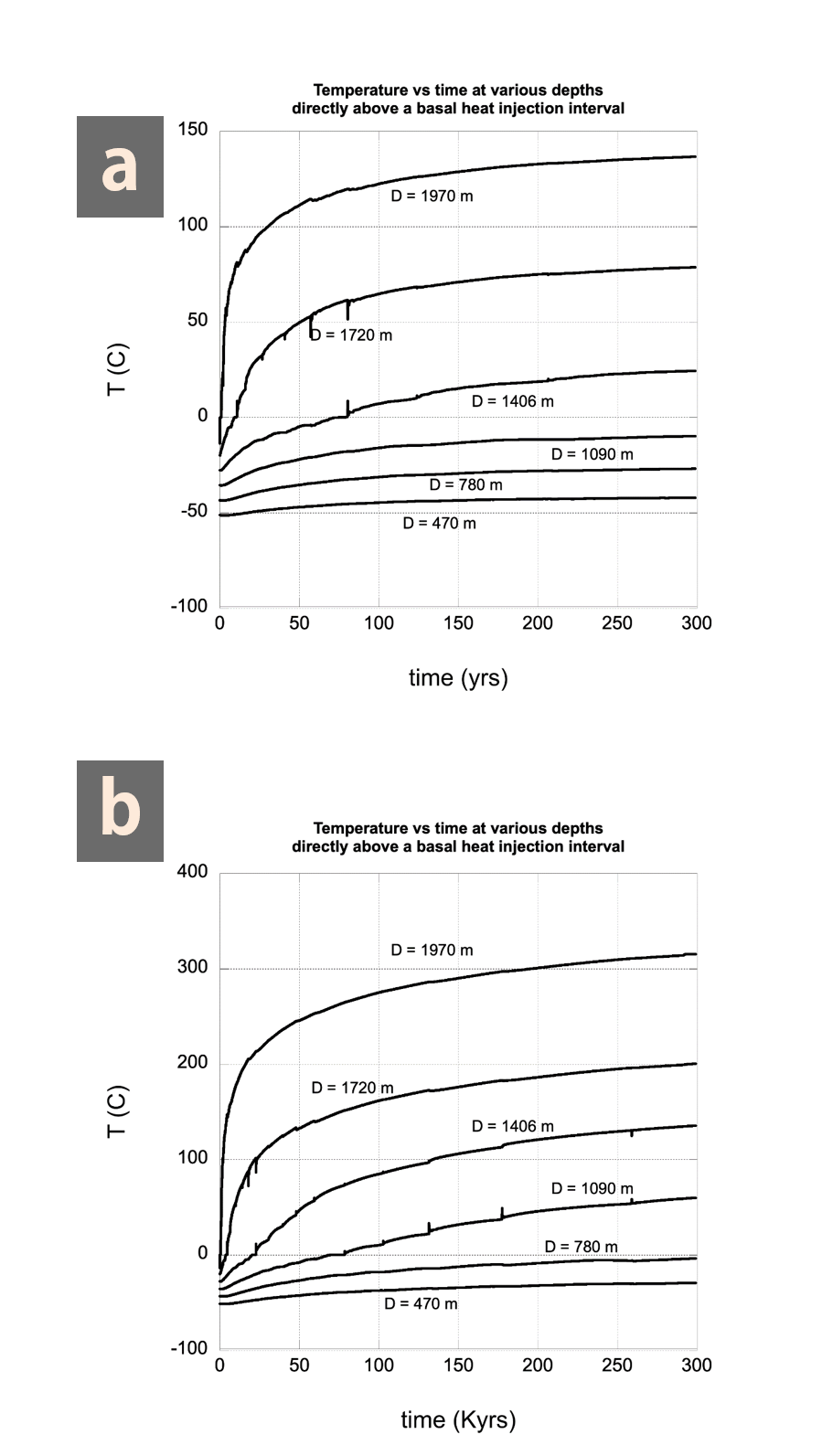


**Figure S7** This figure consists of two plots presenting temperature against time for various depths along a vertical transect through the middle of a basal heating segment. These plots respectively correspond to two distinct heating rates: 1W/m² (as illustrated in Fig. 7e) and 2W/m² (as illustrated in Fig. 7f). It is of particular interest to note that a more extensive domain within the 0-100°C temperature range is evident under conditions of lower heat flow.

**References**

1 Christensen, P. R., Gorelick, N. S., Mehall, G. L. & Murray, K. C. THEMIS Public Data Releases, Planetary Data System node, Arizona State University, <http://themis-data.asu.edu>. (2006).

2 Dickson, J. L., Kerber, L. A., Fassett, C. I. & Ehlmann, B. L. A global, blended CTX mosaic of Mars with vectorized seam mapping: a new mosaicking pipeline using principles of non-destructive image editing, Lunar Planet. Sci. Conf. 49, abstract 2480. http://murray-lab.caltech.edu/CTX/. (2018).

3 Hartmann, W. K. Martian cratering 8: Isochron refinement and the chronology of Mars. *Icarus* **174**, 294-320 (2005). https://doi.org:https://doi.org/10.1016/j.icarus.2004.11.023

4 Michael, G. G. Planetary surface dating from crater size–frequency distribution measurements: Multiple resurfacing episodes and differential isochron fitting. *Icarus* **226**, 885-890 (2013). https://doi.org:https://doi.org/10.1016/j.icarus.2013.07.004

5 Hartmann, W. K. & Daubar, I. J. Martian cratering 11. Utilizing decameter scale crater populations to study Martian history. *Meteoritics & Planetary Science* **52**, 493-510 (2017). https://doi.org:https://doi.org/10.1111/maps.12807
